# Monocyte uptake of polymeric peptidoglycan is bimodal and governed by complement C3 and C4 opsonins

**DOI:** 10.1172/jci.insight.186346

**Published:** 2024-12-10

**Authors:** Narcis I. Popescu, Jędrzej Kluza, Megan A. Reidy, Elizabeth Duggan, John D. Lambris, Linda F. Thompson, K. Mark Coggeshall

**Affiliations:** 1 Arthritis & Clinical Immunology Research Program, Oklahoma Medical Research Foundation, Oklahoma City, Oklahoma, USA.; 2 Department of Pathology and Laboratory Medicine, Perelman School of Medicine, University of Pennsylvania, Philadelphia, Pennsylvania, USA.

**Keywords:** Immunology, Infectious disease, Bacterial infections, Cellular immune response, Complement

## Abstract

Peptidoglycans (PGNs) are structural polymers of the bacterial cell wall and a common microbial molecular pattern encountered by the immune system daily. Low levels of PGNs are constitutively present in the systemic circulation in humans and rise during inflammatory pathologies. Since all known PGN sensors are intracellular, PGN internalization is a prerequisite for the initiation of cellular immune responses. Here, we report the mechanisms controlling the recognition and uptake of polymeric PGNs by circulating human mononuclear phagocytes. We found that complement C3 and C4 opsonins govern PGN recognition and internalization, but no single opsonin is indispensable because of multiple uptake redundancies. We observed a bimodal internalization of polymeric PGNs with distinct requirements for complement C4. At low PGN concentrations, C3 mediated PGN recognition by surface receptors while the efficient internalization of PGN polymers critically required C4. Supraphysiologic PGN concentrations triggered a secondary uptake modality that was insensitive to C4 and mediated instead by C3 engagement of complement receptors 1 and 3. To our knowledge, this is the first description of nonoverlapping C3 and C4 opsonophagocytoses working in parallel. Controlling these uptake mechanisms has the potential to modulate PGN clearance or the dysregulated immune responses during bacterial infections.

## Abstract 2

Monocytes utilize C3 and C4 complement receptors to capture and ingest polymeric peptidoglycans, the hard shell of bacteria.

## Graphical abstract

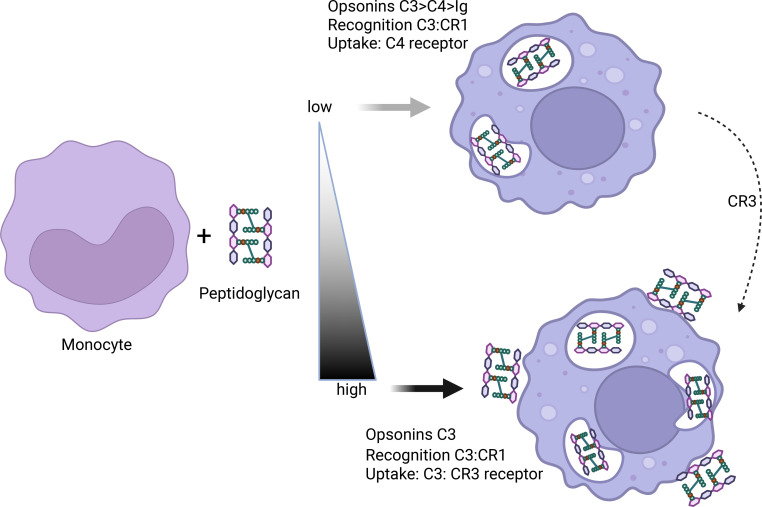

## Introduction

Peptidoglycans (PGNs), linear glycan polymers cross-linked by short polypeptide bridges, are present in virtually all bacteria (1) and comprise some of the most frequently encountered microbe-associated molecular patterns (MAMPs). A steady translocation of PGNs or PGN-derived muropeptides from the commensal gut microbiome (2) leads to stable homeostatic levels of PGN in circulation and peripheral organs (2–4). PGNs interact with pathogen recognition receptors and trigger cellular responses through cytosolic nucleotide-binding oligomerization receptors 1 and/or 2 (NOD1/2) and the inflammasome (5–7). The structural requirements for activation of these receptors are distinct: NOD1 detects diaminopimelate-containing muropeptides (5), NOD2 senses phosphorylated muramyl-dipeptides (MDPs) (6), and the inflammasome is activated by acetylated glucosamines (7) within PGN degradation products. Homeostatic PGN/NOD signaling modulates diverse physiologic functions (8–10), while elevated levels of PGN associate with, and promote, inflammatory pathologies (3, 11–13).

Within the immune compartment, NOD signaling modulates basal and emergency hematopoiesis (14, 15), supports the development of secondary lymphoid structures (16), primes innate (17) and adaptive immune responses (18, 19), and regulates the lifespan of innate phagocytes and lymphocytes (10, 20). Immune responses to PGN are dose dependent (21) and influenced by the structure of muropeptides (22), the ability of immune cells to process NOD ligands (6, 21), and the relative expression of intracellular sensors (23). In humans, circulating innate phagocytes primarily express NOD2 (19, 21, 23, 24), while tissue-resident phagocytes and lymphocytes display a more balanced (23) or NOD1-skewed expression (19, 21, 25). Broadly, PGN/NOD signaling promotes proinflammatory and procoagulant innate immune responses similar to other bacterial MAMPs, such as lipopolysaccharides (22, 26–28), albeit with slower kinetics (27), likely due to requirements for internalization and lysosomal processing of PGN (21). Whereas we know more of PGN/NOD downstream outcomes, the upstream events controlling the immune cell recognition and uptake of PGNs and muropeptides are less understood.

At steady state, circulating immune cells encounter low levels of PGN-derived muropeptides translocated from the gut microbiome (2) that are not enough to trigger inflammatory responses by themselves but prime immune cell reactivity for additional pathogen encounters (17). During dissemination of bacterial infections, however, innate phagocytes encounter both muropeptides and polymeric PGNs because of remodeling of the cell wall during bacterial growth, the release of PGN-containing membrane vesicles from bacteria, and the extracellular killing of pathogens by neutrophils and complement (29). We and others have shown that polymeric PGNs have a higher proinflammatory potential than hydrolyzed muropeptides (26, 30), and the immune responses to PGN polymers mimic bacterial encounters (27, 28, 31). Unlike muropeptides (30), challenges with polymeric PGNs recapitulate virtually all pathophysiological consequences of exposure to parental bacteria, including sepsis progression (28, 31–33). It is therefore important to understand the mechanisms controlling the recognition and internalization of polymeric PGNs to minimize the immune dysfunction during bacterial sepsis.

In contrast with muropeptides, which utilize solute carrier transporters for translocation into the cytosol (34, 35), PGN polymers require serum opsonization for optimal uptake (36–38) and phagolysosomal digestion (21) before the cytosolic transport of NOD agonists. Peptidoglycan recognition protein 1 is a circulating (39) pattern recognition molecule that aids polymeric PGN interaction with immune cells presenting the triggering receptor expressed on myeloid cells 1 (TREM1) (40). TREM1 triggers proinflammatory responses and, at least in neutrophils, enhances the phagocytic activity (41). In addition, we reported that Ig-opsonized PGNs can be internalized by the Fcγ receptor (FCGR) IIa (36). However, through systematic manipulation of serum opsonins and neutralization of opsonophagocytic receptors, we now show that FCGRs are not preferred for monocytes’ uptake of PGN, and they are utilized under limited conditions only. Instead, complement opsonization drives PGN uptake in primary human monocytes through overlapping functions of complement receptors CR1 and CR3. Furthermore, we identify 2 opsonophagocytic modalities for PGN internalization that are governed by C3 and C4 and are differentially employed for PGN uptake at low and high concentrations. Controlling the interactions between these opsonins and their cognate receptors has the potential to improve innate immune responses during acute or chronic inflammatory conditions characterized by elevated circulating PGNs.

## Results

### Ig-mediated opsonophagocytosis is not the preferred pathway for polymeric PGN uptake by primary human monocytes.

**Figure 1:**
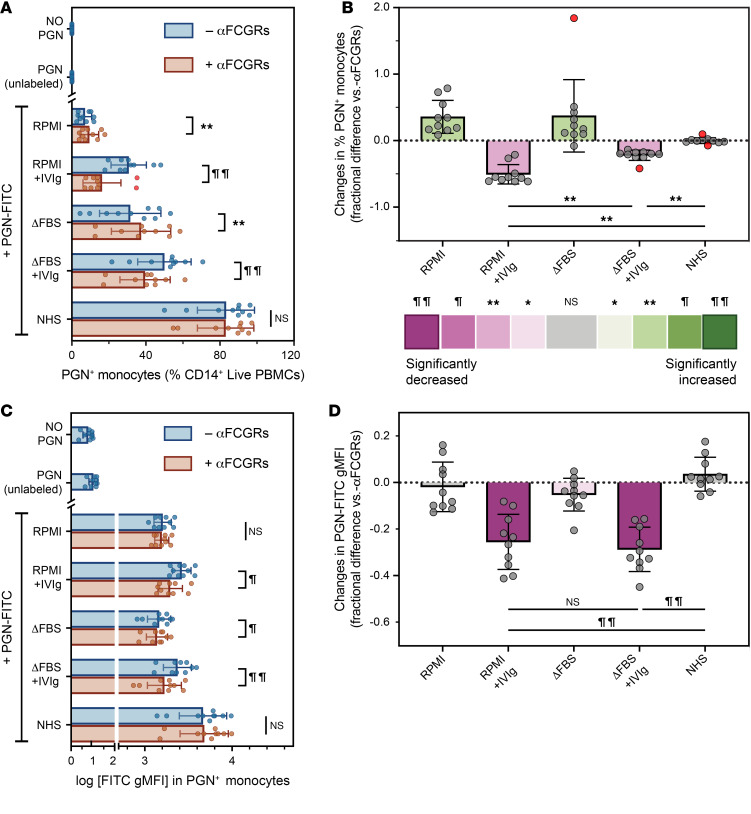
The role of FCGRs during PGN uptake by primary human monocytes. FITC-labeled PGN particles were opsonized, as noted in figure panels, before incubation with PBMCs that were either left untreated (– αFCGRs) or pretreated with FCGR neutralizing Fabs (+ αFCGRs). Changes in the frequencies of PGN^+^ monocytes are depicted before (**A**) or after pairwise normalization to uptake occurring in the absence of FCGR Fabs (**B**). Changes in FITC intensities in PGN^+^ monocytes are shown before (**C**, log-transformed gMFI) or after pairwise normalization to uptake in the absence of FCGR Fabs (**D**). Autofluorescence of unstimulated monocytes (NO PGN) and/or after stimulation with unlabeled PGN are shown for comparison. Data depict individual responses (circles, *n* = 10), group averages ± SD (bars and whiskers), and potential outliers identified by the Tukey’s method highlighted in red. (**A** and **C**) Differences between groups were assessed by RM 2-way ANOVA with Holm-Šídák correction for multiple comparisons. (**B** and **D**) After normalization, deviations from 0, denoting statistically significant effects linked with FCGR neutralization, were assessed by Wilcoxon’s signed-rank test (**B**) or 1-sample *t* test (**D**) and are color-embedded within bars according to the legend. Additional comparisons were assessed by Wilcoxon’s matched pairs signed-rank test (**B**) or paired 2-tailed *t* tests (**D**) and displayed graphically (*P <0.05; **P < 0.01; ^¶^*P* < 0.001; and ^¶¶^*P* < 0.0001). gMFI, geometric mean fluorescence intensity; IVIg, intravenous Ig; RM, repeated measures.

Both polymeric PGNs and hydrolyzed muropeptides require internalization and endolysosomal processing to reach their cognate NOD1, NOD2, and inflammasome receptors (21). The current model for polymeric PGN uptake centers on IgG opsonization and FCGR-mediated phagocytosis (36), yet multiple attempts to control PGN internalization through pharmacologic inhibition of FCGRs have failed in our hands (NIP, unpublished observations). We therefore sought to identify the molecular mechanisms for PGN uptake in primary circulating innate phagocytes through systematic manipulation of serum opsonins and receptor neutralization studies. To this end, freshly prepared human PBMCs were incubated with FITC-labeled PGNs that were either left unopsonized (RPMI) or preopsonized with Igs in the presence or absence of increasing amounts of serum opsonins, either a low-complement (42) fetal bovine serum depleted of bovine Igs (ΔFBS) or pooled normal human serum (NHS) containing all circulating opsonins (Figure 1 and Supplemental Figure 1; supplemental material available online with this article; https://doi.org/10.1172/jci.insight.186346DS1). Serum opsonins had a profound effect on PGN uptake by primary human monocytes. The presence of opsonins resulted in a stepwise increase in the frequency of monocytes taking up PGN (FITC^+^CD14^+^ live PBMCs indicated as PGN^+^ monocytes, Figure 1A) from 7.1% ± 1.1% mean ± SEM in the absence of any opsonin (RPMI) to 31.5% ± 5.2% after ΔFBS and 83.3% ± 4.9% after NHS opsonization (*P* ≤ 0.0027, repeated measures [RM] 2-way ANOVA). Consistent with the current model of polymeric PGN uptake, human Ig (intravenous Ig, IVIg) significantly increased the recognition of PGN in the absence of other opsonins (RPMI + IVIg, Figure 1A), resulting in a 4-fold increase in PGN^+^ monocytes (30.7% ± 3.0% vs. 7.1% ± 1.1%, *P* < 0.0001). Likewise, the amount of PGN taken up, quantified by the FITC fluorescence intensity of PGN^+^ monocytes (Figure 1C), increased by 66%. Similarly, addition of IVIg to low-complement serum (ΔFBS) increased the frequency of PGN^+^ monocytes (31.5% ± 5.2% in ΔFBS vs. 50% ± 4.6% in ΔFBS + IVIg, *P* = 0.0004) and the intensity of monocyte PGN-FITC by 65%. Furthermore, PGN recognition and uptake were sensitive to FCGR neutralization (+αFCGR) whenever IVIg was present. Unexpectedly, however, FCGR utilization gradually decreased with rising concentrations of complement opsonins (Figure 1B). FCGR neutralization inhibited PGN recognition the most when Igs were the only opsonins available (RPMI + IVIg, ~50% reduction), had reduced effects in the presence of low complement (ΔFBS + IVIg, ~22% reduction), and did not influence PGN recognition and uptake in NHS, when high levels of complement opsonins were present (Figure 1, B and D). Our findings indicate that, in contrast with the current model, FCGR-independent mechanisms are favored for the uptake of serum-opsonized PGNs by primary human monocytes.

### Optimal uptake of polymeric PGN requires opsonization by complement C3.

**Figure 2:**
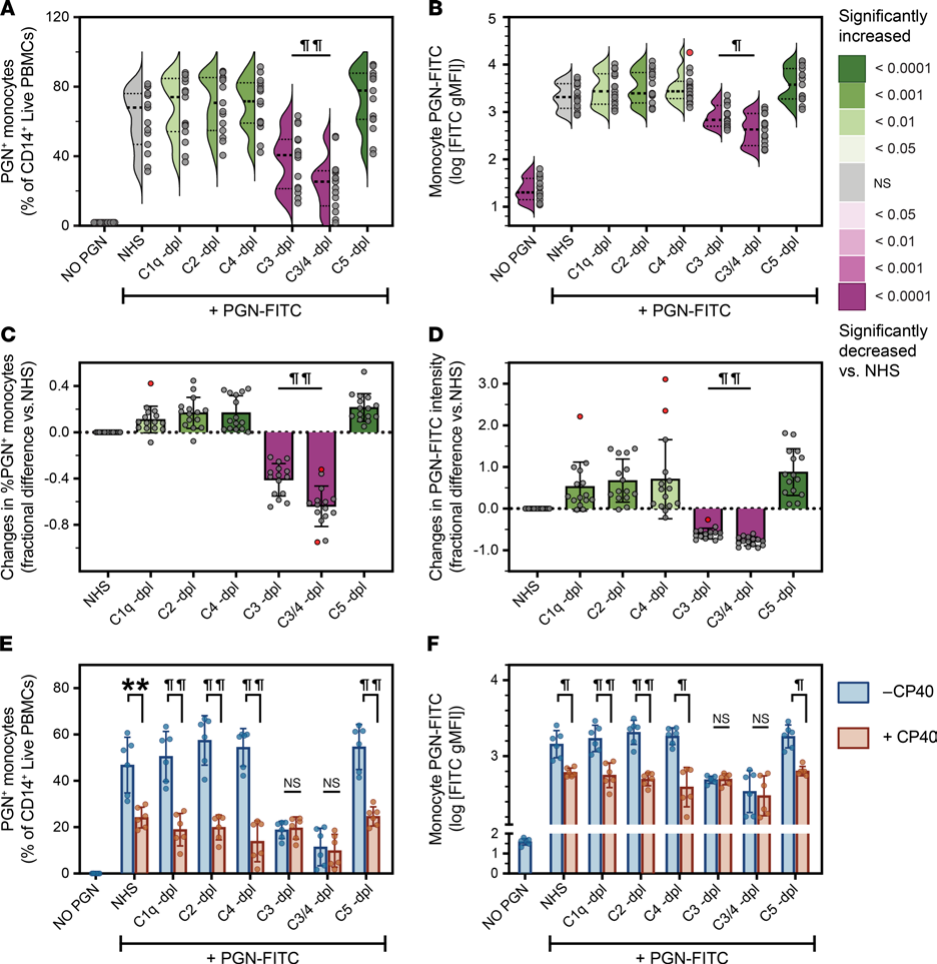
Optimal PGN uptake requires opsonization by complement C3. PGN-FITC particles were preopsonized with either pooled NHS or sera depleted of specific complement factors before incubation with PBMCs. Changes in the frequencies of PGN^+^ monocytes or monocyte PGN-FITC intensities are depicted before (**A** and **B**) or after pairwise normalization to uptake of NHS-opsonized PGN (**C** and **D**). (**A** and **B**) Individual (circles, *n* = 15) and predicted distribution of biological responses (half-violin plots) are depicted, and significant deviations from uptake of NHS-opsonized PGN, assessed by RM 1-way ANOVA with Holm-Šídák correction, are color-embedded according to the legend. (**C** and **D**) Normalized individual and mean ± SD (bars and whiskers) changes due to depletions of complement factors are shown, with potential outliers highlighted in red. Deviations from 0, denoting statistically significant effects associated with complement depletions, were assessed by Wilcoxon’s signed-rank test and color-coded according to the legend. Additional pairwise comparisons between groups were assessed by paired 2-tailed *t* tests and highlighted graphically. (**E** and **F**) PGN-FITC was preopsonized with NHS or sera depleted of specific complement factors in the absence (– CP40) or presence (+ CP40) of 20 μM CP40, an inhibitor of C3 convertases, before incubation with PBMCs. Data depict individual (*n* = 6) and group averages ± SD (bars and whiskers) for the frequencies of PGN^+^ monocytes (**E**) and the intensities of monocyte PGN-FITC (**F**, log-transformed FITC gMFI). Differences between groups were analyzed by RM 2-way ANOVA with Holm-Šídák correction for multiple comparisons and depicted graphically (**P < 0.01; ^¶^*P* < 0.001; and ^¶¶^*P* < 0.0001). -dpl, depleted.

Polymeric PGNs are potent complement activators, both ex vivo and in vivo in nonhuman primates (31, 43), and CRs can support opsonophagocytosis (44). We therefore tested whether PGN recognition and uptake are also mediated by complement. Using human sera immunodepleted of specific complement factors, we found that monocyte recognition and uptake of polymeric PGN were drastically inhibited by the absence of complement C3 (Figure 2 and Supplemental Figure 2). Either C3 depletion (C3-dpl) or C3/C4 double depletion (C3/4-dpl) significantly reduced both the frequency of PGN^+^ monocytes (by 41% and 64%, respectively; Figure 2, A and C) and the fluorescence intensity of PGN-FITC in monocytes (by 60% and 79%, respectively; Figure 2, B and D). No other upstream opsonin tested (C1q, C2, C4), or the downstream C5, reduced PGN recognition and uptake when depleted. Interestingly, combined depletion of C3 and C4 was more effective in reducing PGN uptake than C3 depletion alone (Figure 2, C and D), even though C4 depletion by itself did not diminish PGN uptake. These data indicate that C3 is the primary opsonin for PGN uptake and suggest that C4 may act as an alternate opsonin, whose role is masked by the more potent C3. Alternatively, the loss of C4 could also reduce the activation of the trace amounts of C3 still present in the C3/C4 double-depleted sera. Exhaustive depletions of both C3 and C4 shifted PGN uptake toward FCGR-mediated phagocytosis (Supplemental Figure 2G), providing a redundant mechanism for PGN uptake in primary human monocytes.

To validate immunodepletion studies while maintaining opsonin levels constant, we investigated the effects of pharmacological inhibition of C3 opsonization on PGN-FITC uptake. CP40 is a cyclic peptide inhibitor of C3 convertases that was previously used to block C3 activation both in vitro and in non-human primates (45). In our assays, CP40 reduced PGN recognition and uptake in a dose-dependent manner, with a relative IC_50_ of 1.29 ± 0.07 μM (Supplemental Figure 2, D and E). At saturating inhibitor concentration (20 μM, ~15× IC_50_), CP40 reduced PGN recognition and uptake after opsonization by NHS and all complement depleted sera except, as expected, C3 and C3/C4 immunodepletions (Figure 2, E and F). In the presence of CP40, neither the frequencies of PGN^+^ monocytes (Figure 2E), nor the intensities of monocyte PGN-FITC (Figure 2F), were significantly different between NHS and C1q, C2, C4, and C5 depletions. These findings suggest that the apparent improvements in PGN recognition and uptake observed in the presence of C1q-, C2-, C4-, and C5-depleted sera (Figure 2, A–D) are likely due to variations in C3 levels between these immunodepleted sera and validate C3 as the critical mediator for PGN uptake by primary human monocytes.

### Monocyte opsonophagocytosis of polymeric PGN is mediated by CR1 and CR3.

**Figure 3:**
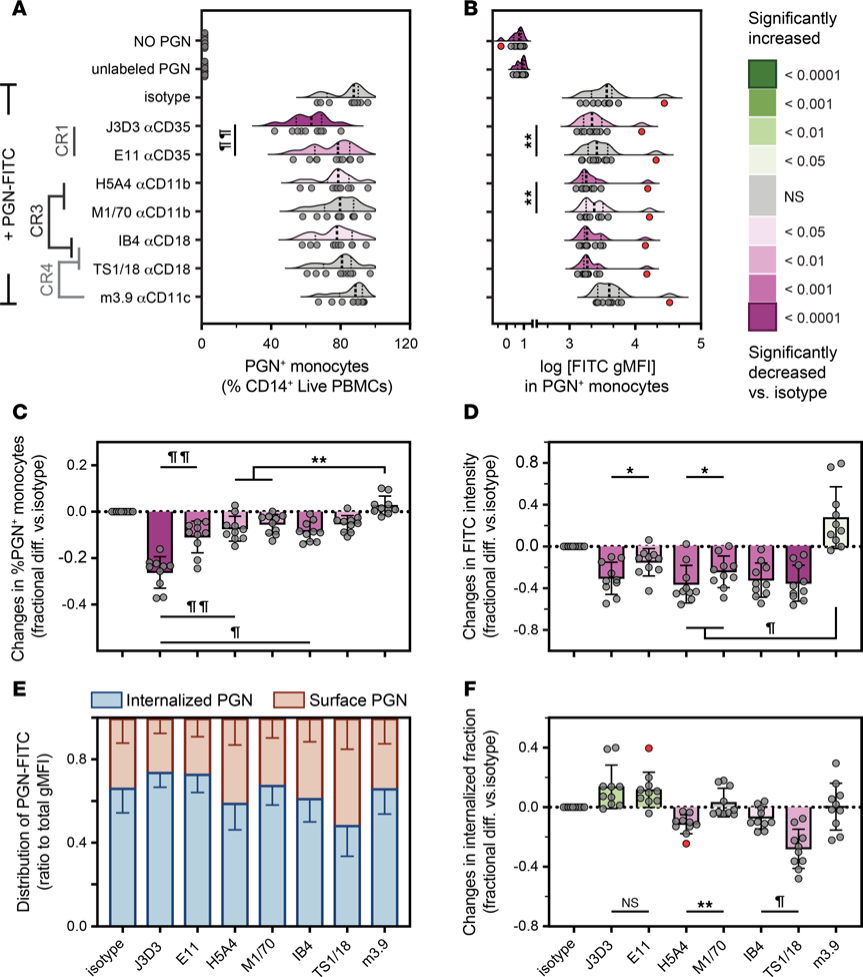
Monocyte opsonophagocytosis of polymeric PGN is mediated by complement receptors CR1 and CR3. Monocytes were preincubated with monoclonal antibodies against CRs before incubation with NHS-opsonized PGN-FITC. Data depict observed frequencies of PGN^+^ monocytes (**A**), FITC intensities in PGN^+^ monocytes (**B**, log-transformed gMFI), as well as changes in frequencies of PGN^+^ monocytes (**C**) and PGN-FITC intensities (**D**) after pairwise normalization to uptake by isotype-treated PBMCs. Individual (circles, *n* = 10) and the predicted distribution of biological responses (half-violin plots) are visualized, with potential outliers depicted in red. (**E** and **F**) Subcellular distribution of PGN was assessed after trypan blue quenching of surface PGN-FITC, and relative proportions are visualized after ratiometric normalization to total cell-associated PGN-FITC. Data are visualized as stacked bars (**E**) depicting mean ± SD (*n* = 10) relative proportions of surface and/or internalized PGN fractions per experimental group, and normalized changes in the internalized PGN are depicted as fractional difference compared with isotype-treated controls (**F**). Differences between groups before normalization were assessed by Friedman’s tests (**A** and **B**). After normalization, deviations from 0 (isotype) were assessed by 1-sample *t* tests (**C** and **D**) or Wilcoxon’s signed-rank test (**F**). Significant differences versus isotype controls are color-embedded according to the legend, while additional pairwise comparisons of interest are depicted graphically (*P < 0.05; **P < 0.01; ^¶^*P* < 0.001; and ^¶¶^*P* < 0.0001).

Complement activation on unprotected surfaces leads to deposition of the C4b and C3b opsonins, which in turn are recognized by CRs. Human monocytes constitutively express multiple C3b receptors that can mediate complement-driven opsonophagocytosis, most notably the single-chain CR1 (CD35) and the β2 integrin heterodimers CR3 (CD11b/CD18) and CR4 (CD11c/CD18) (44, 46). We used neutralizing antibodies against either CR1 (47, 48) or CR3/4 subunits (49–51) to assess the relative contributions of these receptors during polymeric PGN recognition and uptake by human monocytes (Figure 3 and Supplemental Figure 3). PGN uptake was inhibited by multiple neutralizing antibodies targeting CRs albeit none as strongly as C3 opsonin depletion. CR1 antibodies, most prominently J3D3, were the most effective in reducing the frequency of PGN^+^ monocytes (Figure 3, A and C), which we used as a measure of PGN recognition. Inhibition of CR3, using blocking antibodies against either CD11b or CD18, effectively decreased PGN-FITC intensity (Figure 3, B and D) but had a smaller impact on the frequency of PGN^+^ monocytes (Figure 3, A and C). We conclude that CR3 is dispensable for PGN recognition but either stabilizes PGN-receptor complexes on the cell surface or assists with internalization of PGN. Unlike CD11b antibodies, mAb 3.9 against CD11c did not diminish and surprisingly increased the amount of PGN-FITC taken up by monocytes (Figure 3D), suggesting antagonistic roles for CR3 and CR4 during PGN uptake.

We used trypan blue quenching of surface-exposed FITC to interrogate PGN internalization and assess the relative distribution of surface versus intracellular PGN after uptake by monocytes (Figure 3, E and F). CR1 inhibition with either J3D3 or E11 resulted in an apparent shift toward a higher internalized fraction of PGN, indicating a more efficient uptake through CR1-independent mechanisms. In contrast, CR3 inhibition, using H5A4 against CD11b or either of the 2 CD18 mAbs (IB4 and TS1/18), reduced the internalization and enhanced the surface retention of PGN, validating CR3’s involvement in PGN uptake. Importantly, none of these antibodies completely blocked PGN uptake, indicating that redundant PGN internalization mechanisms exist after complement opsonization and utilize at least CR1 and CR3.

### Overlapping roles for CR1 and CR3 during monocyte opsonophagocytosis of polymeric PGN.

**Figure 4:**
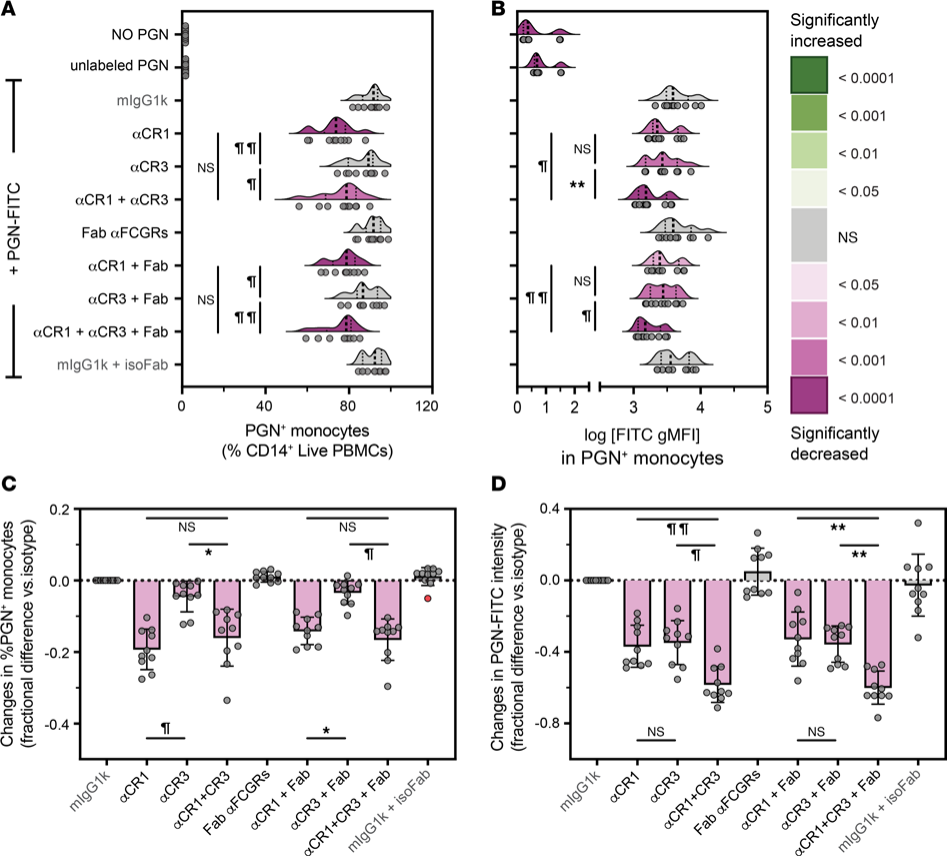
Overlapping roles for CR1 and CR3 during monocyte opsonophagocytosis of polymeric PGN. Monocytes were preincubated with monoclonal antibodies against CR1 (J3D3), CR3 (TS1/18), and FCGRs (Fab), by themselves or in combination, before incubation with NHS-opsonized PGN-FITC. Data depict observed frequencies of PGN^+^ monocytes (**A**), FITC intensities in PGN^+^ monocytes (**B**, log-transformed FITC gMFI), as well as changes in frequencies of PGN^+^ monocytes (**C**) and PGN-FITC intensities (**D**) after pairwise normalization to uptake by isotype-treated PBMCs (mIgG1k). (**A** and **B**) Individual (circles, *n* = 10) and the predicted distribution of biological responses (half-violin plots) are illustrated. Differences between groups before normalization were assessed by RM 1-way ANOVA with Holm-Šídák correction for multiple comparisons. (**C** and **D**) After normalization, data are depicted as group averages ± SD (bars and whiskers, *n* = 10) as well as individual responses (circles), with potential outliers depicted in red. Deviations from 0, denoting statistically significant differences compared with isotype-treated cells, were assessed by Wilcoxon’s signed-rank test and color-embedded according to the legend. Additional pairwise comparisons between groups were assessed by Friedman’s tests and/or RM 1-way ANOVA and highlighted graphically (*P < 0.05; **P < 0.01; ^¶^*P* < 0.001; and ^¶¶^*P* < 0.0001).

To investigate the potential cooperation between CR1 and CR3 during PGN uptake, we preincubated PBMCs with the most potent antibodies against these receptors, J3D3 and TS1/18, by themselves or in combination (Figure 4 and Supplemental Figure 4). Once again, J3D3 inhibition of CR1 resulted in a more prominent loss of PGN^+^ monocytes (19.3% ± 1.8% reduction; Figure 4, A and C) than TS1/18 inhibition of CR3 (4.5% ± 1.4% reduction, *P* = 0.0002 compared with J3D3, Friedman’s test). After the combined inhibition of CR1 and CR3, the frequencies of PGN^+^ monocytes were not statistically different compared to CR1 neutralization alone (Figure 4, A and C), validating CR1 as the main C3b receptor involved in PGN recognition. In contrast, the amount of PGN-FITC taken up by monocytes was similarly reduced by J3D3 anti-CR1 and TS1/18 anti-CR3 (36.8% ± 3.7% and 34.8% ± 3.9% inhibition, respectively, Figure 4D). PGN-FITC intensities were significantly reduced after the combined inhibition of CR1 and CR3 compared with either neutralization alone (58.2% ± 3.2% inhibition, *P* ≤ 0.0002, RM 1-way ANOVA) but less than their additive effects (71.6% ± 6.6% estimated reduction, *P* = 0.008, paired 2-tailed *t* test). Together, CR neutralization studies indicate overlapping roles for CR1 and CR3, whereby PGN capture is primarily mediated by CR1 while the amount of PGN taken up by monocytes is influenced by both CR1 and CR3.

Given the reported cooperation between CR1 and FCGRs (52) and the role of FCGRs in supporting PGN uptake in the absence of complement opsonins (Figure 1), we investigated whether FCGRs could facilitate internalization when either 1 or both CRs were inhibited. Once again, FCGR inhibition alone had no effect and did not enhance the outcomes of CR1 and CR3 neutralizations (Figure 4, C and D). These findings verify that in circulating human monocytes the FCGR-mediated polymeric PGN uptake is not the preferred mechanism for internalization whenever complement opsonins are available.

### Distinct opsonophagocytic modalities are employed for uptake at low and high PGN concentrations.

**Figure 5:**
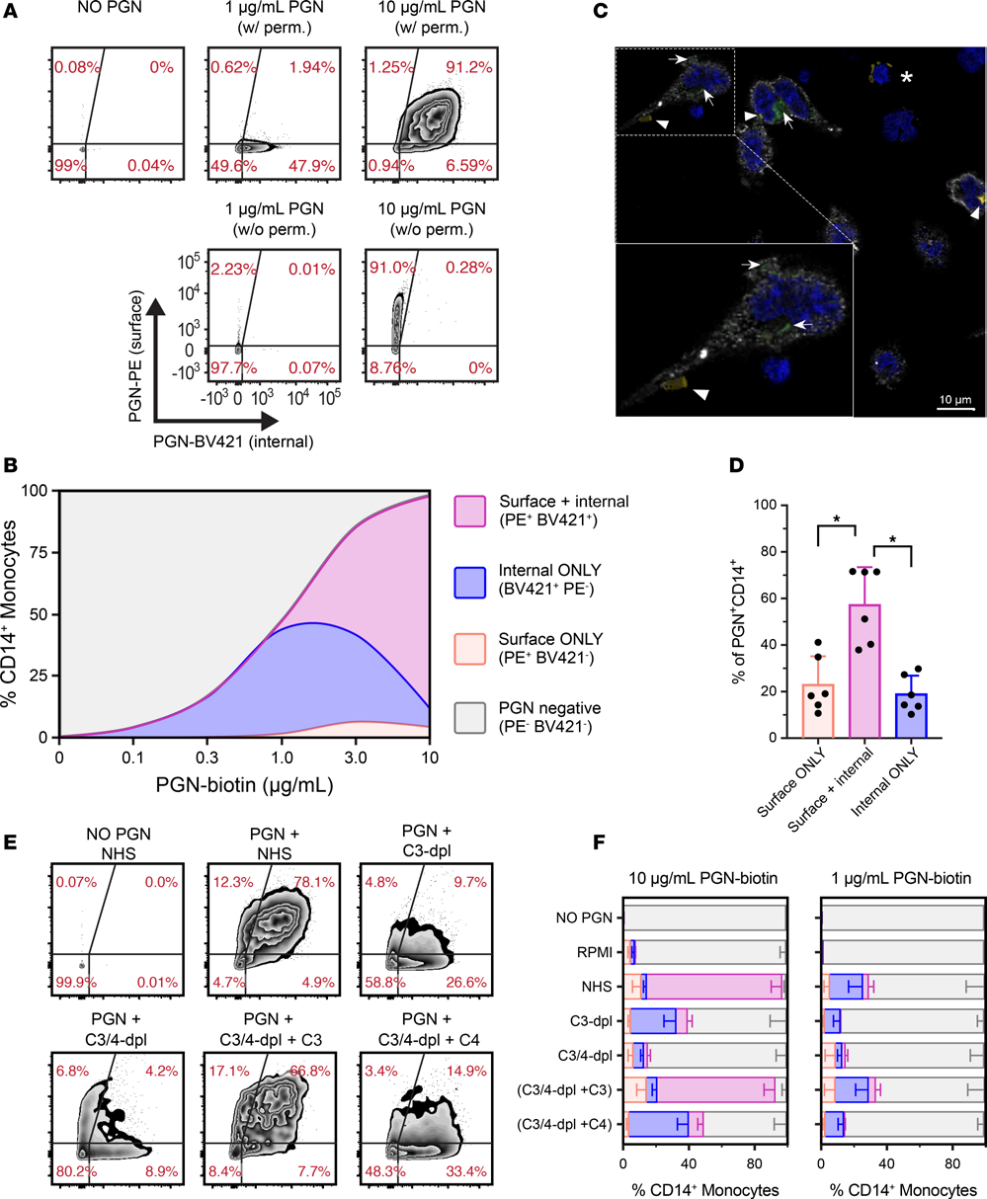
Bimodal internalization of polymeric PGN requires C3 and C4 opsonins. Biotinylated PGN particles were preopsonized with pooled NHS or sera depleted of C3 or C3/C4 opsonins before incubation with human PBMCs. Recognition and uptake of polymeric PGN by primary monocytes were assessed by sequential labeling of surface and internalized PGN using PE- and BV421-labeled streptavidins, respectively. (**A** and **B**) Effect of PGN concentration on uptake of NHS-opsonized polymeric PGN. Representative biaxial plots (**A**) and mean relative distribution of PGN^+^ monocyte subsets (**B**, *n* = 14) containing surface, PE-labeled, and/or internalized, BV421-labeled, polymeric PGN. At low concentrations, PGN-biotin is detected almost exclusively intracellularly, while at high concentrations, most monocytes contain surface and internalized PGN. (**C** and **D**) Confocal analysis of subcellular localization of NHS-opsonized PGN particles (10 μg/mL) after 30 minutes’ exposure to PBMCs. Surface-exposed biotinylated PGN-FITC particles were stained with Alexa Fluor 647–conjugated streptavidin, and the colocalized fluorescent signals were assessed in Imaris (yellow, arrowheads). Internalized PGN-FITC particles were protected from streptavidin staining and detected as FITC fluorescence only (green, arrows). (**C**) Data depict an optical section highlighting monocytes (white, CD14) containing surface and internalized PGN particles. PGN also interacted with, but was not taken up by, CD14^–^ mononuclear cells with rounder nuclei (*). (**D**) Mean relative distribution of PGN^+^ monocyte subsets assessed by confocal microscopy. Data depict group averages ± SD of relative frequencies from 6 donors (circles). RM 1-way ANOVA with Holm-Šídák correction for multiple comparisons (*P < 0.05). (**E** and **F**) Opsonin requirements for PGN recognition and uptake by primary human monocytes. (**E**) Representative biaxial plots, similar to **A**, exemplifying the recognition and uptake of 10 μg/mL PGN-biotin by human monocytes in the presence or absence of C3 and/or C4 opsonins. (**F**) Mean relative distribution of PGN^+^ monocyte subsets after uptake of polymeric PGN preopsonized by NHS or sera depleted of C3 and/or C4. Stacked bars depict group averages ± SD (*n* = 12) of relative frequencies of monocyte subsets, color-coded according to **B**.

To directly evaluate the dynamics of binding and internalization during monocyte uptake of PGN, we performed sequential staining of NHS-opsonized biotinylated PGN using phycoerythrin-labeled (PE-labeled) streptavidin to quantify the surface-retained PGN, followed by cell permeabilization and staining with BV421-labeled streptavidin to quantify the internalized fraction (Figure 5 and Supplemental Figure 5). As previously inferred from functional assays (21, 27), monocyte internalization of PGN was dose dependent (Figure 5B). However, distinct patterns of PGN distribution emerged after internalization at low (≤1 μg/mL) and high (10 μg/mL) PGN concentrations. At 1 μg/mL, we observed an efficient PGN internalization, evidenced by BV421^+^ monocytes lacking surface PGN staining (BV421^+^PE^–^, internalization-only subset; Figure 5, A and B). Under these conditions, PGN retention on the cell surface was minimal, as indicated by the reduced frequencies of PE-labeled monocytes detected as either single-positive PE^+^BV421^–^ (surface-only) or double-positive PE^+^BV421^+^ (surface and internalized) subsets. In contrast, exposure to 10 μg/mL PGN predominantly resulted in PE^+^BV421^+^ monocytes containing both surface and internalized PGN (Figure 5A and Supplemental Figure 5C). Despite variability among donors, the shift toward monocytes having both surface and internalized PGN occurred between 1 and 3 μg/mL PGN in most donors (Figure 5B).

Analysis of PGN fluorescence intensities revealed a surprising dose-dependent shift in the surface PGN intensities among the paired PE^+^ subsets (PE^+^BV421^–^ vs. PE^+^BV421^+^). At low PGN concentrations, as expected, PE intensities were similar or higher on the surface-only (PE^+^BV421^–^) monocytes compared with the paired double-positive subset (Supplemental Figure 5E), consistent with the intracellular translocation of receptor-bound PGN. However, at 10 μg/mL, PGN-PE intensities in the double-positive (PE^+^BV21^+^) subset exceeded those in the surface-only monocytes (Supplemental Figure 5E), suggesting additional engagement of surface receptors at higher PGN levels. The shift toward higher PE staining in the double-positive monocytes correlated with the increased conformational activation of CD18 in this subset (Supplemental Figure 5G), indicating CR3 receptor engagement.

In contrast, the internalized PGN, assessed by BV421 fluorescence, was consistently higher in the PE^+^BV421^+^ monocytes than in the paired BV421^+^PE^–^ subset (Supplemental Figure 5F). At 10 μg/mL, the internalized PGN intensity was 2.7-fold higher in the double-positive subset compared with the internalization-only monocytes. These data indicate that the uptake modality triggered at high PGN concentrations not only facilitates additional capture by surface receptors (Supplemental Figure 5E) but also enhances the internalization of PGN, surpassing the capacity of internalization-only monocytes (Supplemental Figure 5F). The enhanced capture and uptake at high PGN doses are likely mediated by CR3, as CD18 activation was consistently higher in the double-positive monocytes compared with the internalization-only subset (Supplemental Figure 5G).

We conducted a time course analysis of PGN uptake at low (1 μg/mL) and high (10 μg/mL) concentrations to examine uptake progression. Under both conditions, PGN was efficiently internalized within 5 minutes, and the relative distribution of PGN^+^ subsets mirrored the 30-minute endpoint throughout the time course (Supplemental Figure 5H). At low PGN concentrations, PGN^+^ monocytes were predominantly in the internalization-only subset at all time points. Both the internalization-only (BV421^+^PE^–^) and the double-positive (PE^+^BV421^+^) monocytes increased over time (Supplemental Figure 5H), though their ratio remained stable, resulting in a strong positive correlation between them (r*_rm_* = 0.70, *P* < 0.001, repeated-measures correlation, Supplemental Figure 5I). These findings support a progression from internalization-only to double-positive monocytes with repeated PGN encounters. This pattern shifted at high PGN doses. The double-positive subset became dominant at 10 μg/mL and increased over time at the expense of surface-only and PGN^-^ monocytes, showing a strong negative correlation between PE^+^BV421^+^ and PE^+^BV421^–^ subsets (r*_rm_* = –0.79, *P* < 0.001, Supplemental Figure 5J). These results indicate a secondary uptake mechanism activated at high PGN levels, marked by progression from PGN-negative to PE^+^BV421^–^ (surface-only) to PE^+^BV421^+^ monocytes containing surface and internalized PGN.

To verify the subcellular distribution of PGN polymers under these conditions, we analyzed PGN recognition and uptake by peripheral mononuclear cells by confocal microscopy (Figure 5, C and D, and Supplemental Figure 6). FITC-PGN particles were biotinylated for imaging, with extracellular PGN polymers additionally stained using fluorescent streptavidin (Figure 5C, arrowheads). Consistent with flow cytometry results (Figure 5B), PBMCs exposed to 10 μg/mL PGN primarily showed monocytes containing both surface (Figure 5C, arrowheads) and internalized PGN (Figure 5C, arrows) in all donors (*n* = 6, Figure 5D). In addition to CD14^+^ monocytes, PGN also interacted with CD14^–^ mononuclear cells with smaller and rounder nuclei (Figure 5C). These cells bound, but did not internalize, PGN in all instances observed. In line with previous reports (21), we assessed these PGN-interacting cells to be B lymphocytes (Supplemental Figure 7A).

### The role of C3 and C4 opsonins during the bimodal opsonophagocytosis of PGN.

To assess the role of complement opsonins in the observed bimodal uptake of polymeric PGNs, biotinylated PGN was preopsonized with pooled NHS or C3- or C3/C4-depleted sera and incubated with PBMCs, followed by sequential labeling of surface and internalized PGN (Figure 5, E and F, and Supplemental Figure 7). Compared with NHS-opsonized PGN, C3 immunodepletion significantly reduced the frequency of PGN^+^ monocytes at both high (from 97.5% ± 0.4% in NHS to 39.3% ± 2.8% in C3-dpl, *P* < 0.0001, RM 1-way ANOVA) and low PGN concentrations (from 29.0% ± 3.1% in NHS to 11.8% ± 1.2% in C3-dpl, *P* = 0.0007). At the subset level, both surface-only (PE^+^BV421^–^) and double-positive (PE^+^BV421^+^) monocytes were affected. This reduction shifted the PGN^+^ monocytes to the internalization-only subset (BV421^+^PE^–^), similar to the uptake profile seen at low PGN levels (≤1 μg/mL). Additionally, C3 depletion leveled PE intensity between surface-only and double-positive monocytes, and reduced CD18 activation (Supplemental Figure 7, D and F), suggesting decreased CR3 engagement despite exposure to high PGN doses.

Further depletion of C4 (C3/4-dpl) significantly reduced PGN^+^ monocytes at high PGN concentrations (from 39.3% ± 2.8% in C3-dpl to 14.8% ± 1.7% in C3/4-dpl; *P* < 0.0001, RM 1-way ANOVA) but not at low PGN levels. C4 depletion also impaired the intracellular translocation of PGN, as indicated by the reduction in BV421^+^PE^–^ monocytes at both PGN doses (*P* ≤ 0.0025, paired *t* tests). Concurrently, C4 depletion increased the frequency of surface-only monocytes (PE^+^BV421^–^), suggesting that C4 primarily facilitates the internalization of surface-bound PGN polymers rather than their initial recognition. To verify the roles of C3 and C4 opsonins, we reconstituted purified human C3 and C4 into the C3/C4 double-depleted sera during the PGN opsonization step. Adding back C3 alone (C3/4-dpl + C3) restored PGN uptake at low concentrations to levels seen with NHS and recapitulated most of the uptake of NHS-opsonized PGN at high concentrations, despite the reduced C4 (Figure 5, E and F). These findings verify that C3 is essential for PGN recognition and suggest that internalization can occur at very low C4 levels, though possibly at a slower rate, as indicated by higher surface retention of PGN (PE^+^BV421^–^) and lower double-positive monocytes compared with NHS-opsonized cargo. Reintroducing C4 into C3/C4-depleted serum (C3/4-dpl + C4) enhanced PGN uptake and shifted the subset distribution toward internalization-only monocytes. This supports a primary role for C4 in internalization, as C4 reconstitution increased both the internalization-only (*P* ≤ 0.0217, paired 2-tailed *t* tests) and double-positive monocytes (*P* ≤ 0.0163, paired 2-tailed *t* tests) above levels observed with C3-depleted serum.

Overall, these findings indicate a bimodal PGN uptake involving distinct receptor engagements. At low concentrations, C3 mediates PGN recognition and C4 facilitates its internalization, leading predominantly to internalization-only (BV421^+^PE^–^) monocytes. The minimal CD18 activation indicates that CR3 does not significantly contribute to this mechanism. In contrast, at high PGN levels, a secondary CR3-mediated uptake emerges largely independent of C4 and generates double-positive monocytes with both surface-bound and internalized PGN. Both uptake modalities seem monocyte specific as B lymphocytes, CR1-expressing mononuclear cells known to interact with polymeric PGN (21), display only complement-mediated surface binding with no significant PGN internalization under the same experimental conditions (Supplemental Figure 7A).

### The role of CR1 receptor during the bimodal uptake of polymeric PGN.

**Figure 6:**
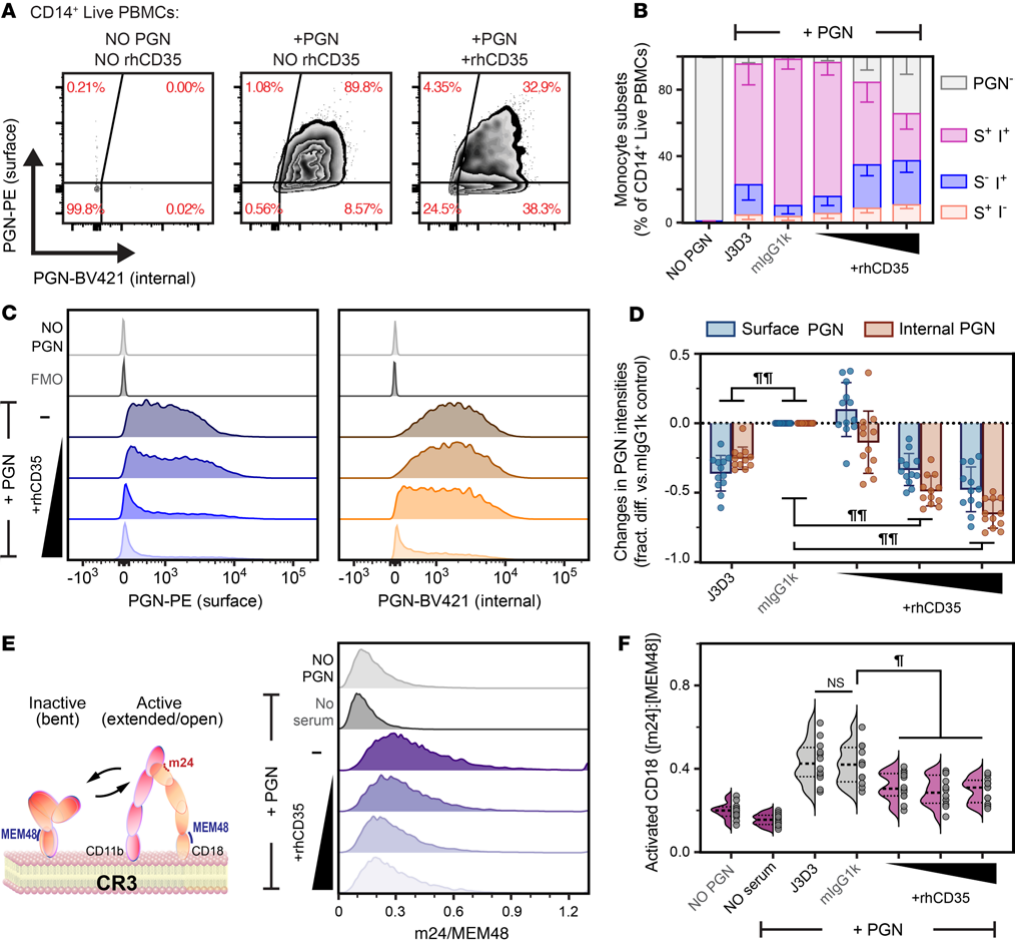
Soluble CR1 (rhCD35) reduces both recognition and internalization of polymeric PGN. Human PBMCs were incubated with 10 μg/mL NHS-opsonized, biotinylated PGN in the presence or absence of CR1 inhibitors, and the surface and internalized PGN fractions were sequentially stained using PE- and BV421-labeled streptavidins. (**A**) Representative biaxial plots exemplifying the effects of soluble CR1 (rhCD35) on PGN uptake by primary human monocytes (see Supplemental Figure 8A for additional plots). (**B**) Changes in the relative distribution of monocyte subsets containing surface and/or internalized PGN after CR1 inhibition using either the J3D3 monoclonal or increasing concentrations of the extracellular domain of CR1 (rhCD35). Data are visualized as stacked bars depicting mean ± SD (*n* = 12) relative frequencies of monocyte subsets per experimental group (legend: S = surface, PE-labeled PGN; I = internalized, BV421-labeled PGN). (**C**) Representative histogram overlays depicting the shift in fluorescence intensities of surface (left panel) and internalized (right panel) PGN in response to rhCD35 treatment. (**D**) Normalized changes in the surface and internalized PGN intensities associated with CR1 inhibition, illustrated as fractional difference compared with an irrelevant challenge (mIgG1k). (**E**) Competitive inhibition of CR1-PGN interaction by rhCD35 reduces the conformational activation of CR3, indicative of a crosstalk between the 2 CRs. Assay principle (left) and representative histogram overlay of CR3 activation depicted as the ratio between 2 CD18 reporter mAbs, the activation-dependent m24 and the activation-insensitive MEM48. (**F**) Changes in CR3 activation associated with CR1 inhibition in the experimental cohort (*n* = 12). Individual (circles) and the predicted distribution of biological responses (violin plots) are illustrated. (**D** and **F**) Differences between experimental groups were assessed by RM 2-way (**D**) or 1-way (**F**) ANOVA with Holm-Šídák correction for multiple comparisons and depicted graphically (^¶^*P* < 0.001; and ^¶¶^*P* < 0.0001). FMO, fluorescence minus one.

CR1 (CD35) is a large, modular complement receptor that can bind either C3b or C4b and potentially mediates both uptake mechanisms. We therefore tested the involvement of CR1 in the PGN uptake through competitive inhibition with recombinant CR1 (rhCD35; Figure 6 and Supplemental Figure 8). rhCD35 reduced the frequency of double-positive (PE^+^BV421^+^) monocytes in a dose-dependent manner and concurrently increased the frequencies of PGN^–^ (PE^–^BV421^–^) and internalization-only (BV421^+^PE^–^) subsets (Figure 6B). Likewise, rhCD35 reduced the fluorescence intensities of both surface and internalized PGNs (Figure 6, C and D). At the highest concentration tested (30 μg/mL), rhCD35 decreased the internalized PGN intensity by approximately 65%, consistent with the higher PGN internalization seen in double-positive monocytes compared with the paired internalization-only subset. Unexpectedly, rhCD35 reduced the conformational activation of CD18 (Figure 6, E and F), indicating a direct crosstalk between CR1 and CR3 during PGN uptake. The anti-CR1 antibody J3D3 had a smaller effect, redistributing monocytes from the double-positive to the internalization-only subset and moderately reducing PGN fluorescence intensities in both subcellular fractions (Figure 6, B and D). These results indicate that CR1 plays a role in generating double-positive monocytes with surface-bound and internalized PGN, likely through coordinated action with CR3. rhCD35 inhibition of CR1 does not eliminate, however, the internalization-only subset, suggesting that CR1 is not the C4b receptor responsible for this uptake.

## Discussion

In this study we have refined the model for the recognition and uptake of polymeric PGNs by primary human monocytes, a critical step in initiating immune responses to this common bacterial MAMP (21). We show that the established model of Ig-mediated, FCGR-driven phagocytosis of polymeric PGN (36) occurs only in the absence of multiple complement opsonins. Instead, we found that complement opsonization that occurs through all 3 initiation pathways (31) leads to deposition of C4b and C3b opsonins, which direct PGN recognition and uptake through CRs. Our findings from opsonin immunodepletions and receptor neutralization studies support this model and reveal a functional overlap between the C3b receptors CR1 and CR3. C3b-opsonized PGNs were preferentially recognized by CR1, while additional engagement of CR3 was triggered at high PGN concentrations (Figures 3 and 5). We found that C4b has a complementary role during PGN uptake, which was highlighted by the absence of C3 (Figure 2) and the inhibition of CR1 with rhCD35 (Figure 6). C4b facilitated the rapid intracellular translocation of PGN at low concentrations and worked in parallel with the higher capacity, CR3-mediated uptake at high doses of PGN. To our knowledge, this study is the first to demonstrate that C4b-mediated opsonophagocytosis works alongside C3b in human innate phagocytes, potentially affecting other complement-opsonized cargoes. Together, our results show that human monocytes employ redundant mechanisms for recognizing and internalizing polymeric PGNs, allowing rapid uptake at low concentrations and reserving CR3 receptors to handle higher PGN loads. These mechanisms appear monocyte specific, as B cells fail to internalize PGN even at high concentrations, despite expressing CR1 and binding PGN through complement-mediated interactions.

Previous studies from our group supported an Ig-mediated model for PGN phagocytosis, requiring FCGR2A, Syk kinases, and actin cytoskeleton remodeling (21, 27, 36–38). Our current findings, however, reveal that CRs outperform FCGRs during polymeric PGN uptake in primary monocytes. Most commonly, PGN recognition was driven by C3b interaction with CR1 and the intracellular translocation of receptor-bound PGN was facilitated by either C4b receptors or iC3b engagement of CR3 at high PGN doses. Although CR3 is a known phagocytic receptor that supports internalization through both sinking and phagocytic cup mechanisms (53), the identity of the C4b phagocytic receptor in monocytes has eluded us so far. Notably, monocytes express Ig-like transcript 4 (ILT4) receptors that bind C4 byproducts (54), but ILT4 involvement in internalization events awaits experimental confirmation. A C3-independent, C4b-mediated phagocytosis through an unidentified C4b receptor was recently reported in astrocytes (55), arguing that this mechanism might be widespread among immune effectors.

Complement-mediated opsonophagocytosis is critical for the clearing of pathogens and MAMPs during infections (44), as well as apoptotic cells and debris during homeostasis (55, 56). Our results show that PGN opsonophagocytosis by human monocytes requires minimal C3 opsonization that can be achieved through any complement pathways. C4 could play a backup role in the absence of C3, and when both C3 and C4 are depleted, PGN uptake shifts toward FCGRs (Supplemental Figure 2G). Although the evolutionary benefits for maintaining multiple redundant mechanisms are not fully obvious, they counteract immune evasion by bacterial pathogens since polymeric PGNs reflect bacterial infections. In contrast, PGN transfer from commensal bacteria across the intestinal epithelia primarily involves smaller muropeptides that do not activate the complement cascade (57) and are therefore unlikely to trigger similar events.

Circulating mononuclear phagocytes express multiple complement receptors, most notably CR1, CR3, and CR4, at levels equal to or exceeding FCGRs (46). All these receptors are multifunctional, and they also present multiple binding sites for complement opsonins (58–60). This may explain why opsonin depletion was always more effective at reducing PGN uptake than any receptor neutralization in our study. Whereas CR1 binds C3b, the integrin CRs engage the iC3b byproduct generated by factor I–mediated (FI-mediated) proteolysis of C3b. Despite structural similarities, CR3 and CR4 engage iC3b through distinct binding sites (60), which could explain their antagonistic effects. Our studies indicate that CR3 engagement of iC3b-opsonized PGN triggers the active CR3 conformation supporting outside-in integrin signaling (61), a critical step during uptake. CR3 neutralization reduced both the conformational activation of the integrin and the uptake of PGN. In contrast, CR4 neutralization had opposite effects, increasing PGN uptake and the conformational activation of CD18, the shared β2 chain. Our findings indicate that CR4 competes with CR3 for iC3b binding, but engagement of iC3b does not activate CR4, which consequently cannot internalize PGN. We conclude that CR3 is the primary uptake receptor, at least at high PGN doses, while CR4 acts as a decoy, especially in nonclassical monocytes that express higher levels of CD11c (46).

CR1 had the largest impact on PGN recognition by primary human monocytes in our studies. CR1 is a polymorphic receptor consisting of 3–6 long homologous regions (LHRs) with a high degree of identity (58). This modular receptor has multiple C3b/C4b binding sites across its LHRs enabling strong interactions with complement-opsonized particles through an intramolecular avidity effect (62–64). Avidity disruptions may explain why J3D3, which binds more LHRs than E11 (47), was more potent in our studies. In line with a higher binding affinity for C3b (63, 64), the role of C4 in PGN recognition became apparent only after C3 depletion (Figures 2 and 5). Besides C3b/C4b (58), CR1 also binds C1q and mannan-binding lectin (65, 66), which could explain the low, but measurable, PGN uptake when both C3 and C4 are depleted. Additionally, CR1 functions as an allosteric cofactor for FI-mediated proteolysis of C4b and C3b, generating iC4b and iC3b fragments, respectively (63, 64).

CR1 and CR3 appear to cooperate during PGN uptake at high doses. CR1, the primary C3b recognition receptor, captures complement-opsonized PGN polymers and amplifies FI-mediated conversion to iC3b necessary to engage CR3/CR4. Consequently, stable intermolecular complexes form between opsonized PGN and both CR1 and CR3 (67), which in turn trigger CR3-dependent internalization. CR1 impairment by monoclonal antibodies or soluble rhCD35 destabilizes these complexes and reduces CR3 engagement and PGN uptake. CR3 can bind iC3b-opsonized PGN independently of CR1, but the likelihood for these encounters is reduced without retention of PGN on the cell surface. At low PGN concentrations, we hypothesize that C4b opsonization mimics iC3b’s role and engages internalization receptors. This would explain the C4 requirements for uptake but not for PGN recognition, where C3b was still preferred (Figure 5 and Supplemental Figure 7), and would argue that CR1 is not an internalization effector. In line with this hypothesis, B lymphocytes bound but did not internalize PGN despite expressing CR1 (46), suggesting that internalization effectors were missing in these cells.

Our study uncovered a dose-dependent shift in PGN uptake pathways through differential employment of internalization receptors. At low PGN concentrations, C4b receptors were extremely efficient at internalizing PGN, leading to almost complete translocation of surface-bound PGN inside monocytes. They also worked in parallel with the higher capacity, iC3b:CR3 uptake triggered by supraphysiologic PGN concentrations. The sequential employment of distinct internalization receptors can be explained by a progressive consumption of C4b receptors with increasing PGN concentrations. As more C4b receptors internalize with cargo, and are therefore lost from the cell surface, the prolonged retention of remaining CR1-captured PGN allows for engagement of integrin receptors. Alternatively, FI mediated proteolysis of both C4b and C3b, which generates iC4b and iC3b, may shift the affinity away from the C4b receptors and toward CR3/CR4. Although additional studies are needed to differentiate between these mechanisms, they ensure that the multifunctional integrin CRs are engaged only when PGN exceeds physiologic thresholds.

In summary, our findings reveal substantial redundancies for monocytes’ recognition and uptake of polymeric PGNs, with backups in place across opsonins, receptors, and endocytic pathways. The efficient C4-mediated uptake at low PGN concentrations allows monocytes to rapidly detect and internalize circulating PGN polymers at physiologically relevant levels (3, 4). Our study supports the idea that genetic variations in C4, CR1, and CR3, associated with autoimmune disease susceptibility (68–70), could disrupt PGN recognition and promote systemic inflammatory conditions (3). In addition, high PGN levels can promote a sepsis-like progression (28, 31, 33) and interventions targeting PGN uptake, such as soluble CR1 competition (71) or CR3 inhibition, may have therapeutic potential in managing severe inflammatory responses during bacterial infections.

## Methods

### Sex as a biological variable.

The current study examined both men and women, and similar findings were observed regardless of sex. Combined data were used for analysis and visualization throughout the manuscript, while sex stratification of biological responses is reported in supplemental figures.

### Reagents, chemicals, and antibodies.

PGN was purified from *Bacillus anthracis* strain Sterne BA781 (Δlef243/Δcya244/ΔpagA242) obtained through the NIH Biodefense and Emerging Infections Research Resources Repository (NR-9401), National Institute of Allergy and Infectious Diseases, according to protocols detailed elsewhere (21, 72, 73). Purified PGN preparations were free of lipoproteins and TLR2/TLR4 ligands and supported internalization-dependent monocyte responses (27) through NOD and inflammasome signaling. The median size of polymeric PGNs, assessed by nanoscale flow cytometry (37), was 240 ± 8 nm. PGN aliquots were amine-labeled using either FITC (MilliporeSigma) or EZ-link Sulfo-NHS-LC-Biotin (Pierce, Thermo Fisher Scientific) for 1 hour at room temperature in 0.1 M carbonate buffer pH 9.0. Particles were collected by centrifugation, quenched with 0.1 M glycine, washed extensively with Dulbecco’s phosphate buffered saline (DPBS), and stored in DPBS at 4°C protected from light.

Ultralow-IgG FBS (ΔFBS), a serum with low complement and extremely low bovine IgG (<0.1 μg/mL), was from Life Technologies, Thermo Fisher Scientific. Human sera immunodepleted of specific complement components, and purified complement proteins, were from Complement Technology. The manufacturer certified depletions at ≥95% of mean plasma levels. CP40, the C3 convertase inhibitor used in the study, has been characterized previously (45). Ig supplementation at 1 mg/mL final concentration was achieved with IVIg (Gamunex-C, 10% Ig, Grifols Therapeutics). For FCGR neutralization we used a mixture of Fab fragments, each at 10 μg/mL, against CD16 (clone 3G8), CD32 (clone 7.3), and CD64 (clone 10.1), all from Ancell. For CR neutralization we used full-length monoclonal antibodies at 30 μg/mL: J3D3 (Beckman Coulter) or E11 (Ancell) against CR1, IB4 (Ancell) or TS1/18 (BioLegend) against CD18, and the 3.9 monoclonal antibody against CD11c (BioLegend). Neutralizing antibodies against CD11b were purified from M1/70.15.11.5.2 (50) or H5A4 (51) hybridomas obtained from the Developmental Studies Hybridoma Bank. Isotype controls included mouse IgG1k Fab (isoFab, clone MOPC31C, Ancell), mouse IgG1k (mIgG1k, clone MOPC21, BioLegend), and rat IgG2b (clone RTK4530, BioLegend). Flow cytometry reagents, detection antibodies, and labeled streptavidins were either from Life Technologies, Thermo Fisher Scientific (clones 61D3 against CD14 and MEM48 against CD18) or BioLegend (clones m24 against CD18 and HIB19 against CD19, PE-labeled [catalog no. 405245] and BV421-labeled [catalog no. 405225] streptavidins, and Zombie Violet [catalog no. 423114] or Aqua [catalog no. 423102] fixable viability dyes).

### Analysis of primary human mononuclear cells.

PBMCs were isolated from heparinized blood by density gradient centrifugation using Histopaque-1077 (MilliporeSigma). Unless otherwise specified, PBMCs were rested in RPMI (Corning Life Sciences) for at least 15 minutes on ice before the addition of preopsonized PGNs. In some experiments, neutralizing antibodies for opsonophagocytic receptors, either FCGRs or CRs, were added to PBMCs for 30 minutes on ice before addition of preopsonized PGNs and kept throughout the internalization experiment. For CR1:C3b competition by the soluble extracellular domain of CR1, rhCD35 (R&D Systems, Bio-Techne) was incubated with preopsonized PGN particles for 30 minutes on ice before addition to PBMCs.

### PGN internalization studies.

Internalization studies followed 2 experimental designs, using either PGN-FITC followed by trypan blue quenching of surface FITC or PGN-biotin followed by sequential staining of surface and internalized PGN fractions using labeled streptavidins. PGNs were preopsonized at 6× concentration in 60% sera, either pooled NHS or sera depleted of specific complement factors, for 30 minutes at 37°C in a water bath, then chilled for at least 15 minutes on ice. Preopsonized PGN was added to chilled PBMCs on ice, and internalization was initiated by transferring reactions to a 37°C water bath and was allowed to proceed for 30 minutes, except for Figure 1 experiments, where the time was extended to 1 hour for comparison with the slower nonopsonic uptake. Afterward, all processing steps were performed on ice to eliminate additional internalization of surface-bound PGN. FITC-PGN internalization, trypan blue quenching of surface-exposed fluorescein, and subsequent postacquisition corrections have been detailed previously (37). For sequential staining of biotinylated PGNs, surface-bound PGN-biotin was stained with 20 μg/mL PE-labeled streptavidin together with viability stains and cell surface markers (CD14 and/or CD19) as detailed in Supplemental Figure 5A. Cells were subsequently washed, fixed with 4% paraformaldehyde, permeabilized with 0.1% saponin, and stained for intracellular PGN using 2 μg/mL BV421-labeled streptavidin in the presence of saponin. To correct for the binding of BV421-streptavidin to putative unoccupied cell surface sites, a nonpermeabilized stain protocol where fixation and saponin were omitted was run in parallel for each experimental condition. Data were acquired on either an LSRII cytometer (BD Biosciences) using FACSDiva software (version 9.0) or on a Cytek Aurora (Cytek Biosciences) spectral cytometer using SpectroFlo software (version 3.3). Postacquisition processing and analyses of flow cytometry data were performed in FlowJo (versions 9 or 10), and individual readouts depict frequencies of PGN^+^ mononuclear cells and gMFIs, usually log-transformed.

### Conformational activation of CR3/4.

To assess the conformational activation of integrin CRs, we used 2 CD18 reporter mAbs with distinct conformational requirements: the activation-dependent m24 (BioLegend) and the activation-insensitive MEM48 (Life Technologies, Thermo Fisher Scientific), which were added to the surface staining cocktail. We normalized activated CD18 (m24 intensity) to total CD18 staining (MEM48 intensity) on a cell-by-cell basis by generating a derived parameter in FlowJo depicting the m24/MEM48 ratio and used the geometric mean of the m24/MEM48 ratio as a discrete global readout of CD18 activation in subsequent analyses.

### Imaging analysis of PGN uptake.

We performed confocal microscopy analysis of PGN internalization to assess the subcellular localization of bound and/or internalized PGNs. Briefly, primary human PBMCs were captured on poly-l-lysine–coated circular coverslips in 24-well plates and incubated for 30 minutes at 37°C with 10 μg/mL NHS-opsonized PGN prelabeled with both FITC and biotin. Cellular processes were stopped by fixation with one-quarter volume of 16% paraformaldehyde for 30 minutes at room temperature. Without permeabilizing the cells, surface-exposed PGN was detected using an Alexa Fluor 647–conjugated streptavidin (Life Technologies, Thermo Fisher Scientific), while monocytes were detected with a mouse mAb against human CD14 (clone 61D3, Life Technologies, Thermo Fisher Scientific) followed by secondary detection using an affinity-purified, cyanine 3–labeled donkey anti-mouse IgG (catalog number 715-165-151, Jackson ImmunoResearch Laboratories). Nuclei were counterstained with DAPI and samples were mounted in ProLong Gold (both from Life Technologies, Thermo Fisher Scientific) and cured for 24 hours at room temperature in the dark. Cells were visualized on a ZEISS LSM 980 multiphoton confocal microscope using a Plan Apochromat 63×/1.40 NA oil immersion objective. *Z*-stack images were collected with 0.21 μm step increments using the Zen acquisition software (version 3.8, Carl Zeiss Microscopy). Confocal datasets were processed postacquisition in Imaris software (version 10.2.0, Oxford Instruments), and micrographs of representative sections are shown. Colocalization of FITC and Alexa Fluor 647 signals, denoting surface-exposed PGN particles, was generated in Imaris after *Z*-stacks were segmented based on CD14 staining to limit analysis to cell-associated PGN. For quantitation, flattened maximum intensity projections of 5–8 independent *Z*-stacks per donor were manually assessed for the presence of internal (FITC only) and surface (colocalized FITC and Alexa Fluor 647) PGN particles in CD14^+^ monocytes (66–234 independent CD14^+^ cells/donor) and depicted as relative proportion of PGN^+^ monocytes.

### Statistics.

Except for the RM correlation analysis performed in R using the *rmcorr* package (74), all statistical analyses were performed in Prism (versions 9.3 to 10.3, GraphPad Software). Normal distribution of biological responses was assessed using Shapiro-Wilk tests and QQ plots. Potential outliers, identified by Tukey’s method, are highlighted for reference but were not excluded from analysis. Differences between 2 groups were assessed by paired 2-tailed *t* tests, for normally distributed datasets, or Wilcoxon’s matched pairs signed-rank test otherwise. Differences between multiple groups were assessed by RM 1- or 2-way ANOVA with Geisser-Greenhouse correction for sphericity and Holm-Šídák multiple comparisons test for normally distributed data or Friedman’s tests otherwise. When pairwise comparisons were hindered by missing values independent of the experimental design (samples lost during processing or acquisition), comparisons between multiple groups were assessed by a mixed effects, restricted maximum likelihood model with Geisser-Greenhouse and Holm-Šídák corrections. For normalization, baseline in the absence of PGN (NO PGN) was subtracted from paired experimental readouts and the reference positive controls, usually uptake of NHS-opsonized PGN in the absence of receptor neutralization unless otherwise specified. To reduce intragroup variability, readouts were scaled to the paired reference uptake for each donor, and the effects of opsonin depletions and/or receptor neutralizations are depicted as fractional difference after ratiometric normalization to the reference. Consequently, after normalization, significant deviations from 0 (normalized reference value) were assessed by 1-sample 2-tailed *t* test, for normally distributed datasets or Wilcoxon’s signed-rank test otherwise. In all statistical tests, *P* values lower than 0.05 were considered significant, and standard significance levels are depicted graphically.

### Study approval.

The study was performed in accordance with the Declaration of Helsinki and approved by the IRB at the Oklahoma Medical Research Foundation (protocol number 19-11). Healthy volunteers, both men and women, provided written informed consent before each blood draw.

### Data availability.

Values for all data points used for visualizations are reported in the Supporting Data Values file.

## Author contributions

NIP and KMC conceptualized the study. JDL provided critical reagents and expertise for complement inhibition. JK, MAR, ED, and NIP performed experiments. NIP curated data and performed formal analysis and visualizations. NIP, LFT, and KMC interpreted results and wrote the manuscript. LFT and KMC acquired funding and administered the project. All authors reviewed and edited the manuscript.

## Supplementary Material

Supplemental data

Supporting data values

## Version 1 12/10/2024

In-Press Preview

## Version 2 01/23/2025

Electronic publication
