# Supplementary material for: Monocyte uptake of polymeric peptidoglycan is bimodal and governed by complement C3 and C4 opsonins
Source: JCI Insight. 2024 Dec 10;10(2):e186346. doi: 10.1172/jci.insight.186346 (PMC11790019; doi:10.1172/jci.insight.186346)
Supplement: Supplemental data [file jciinsight-10-186346-s100.pdf]

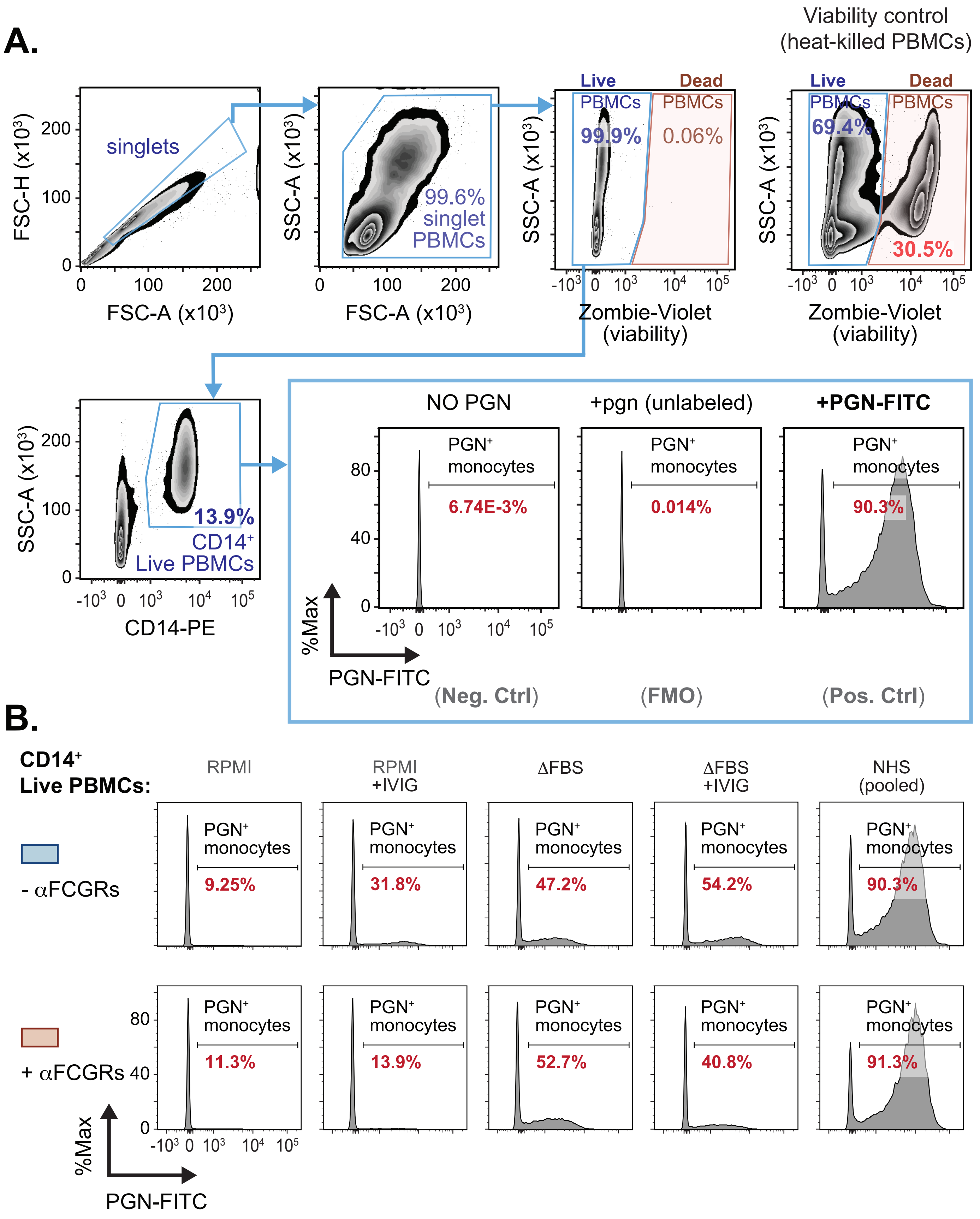

(Supplemental Figure 1 continues next page)

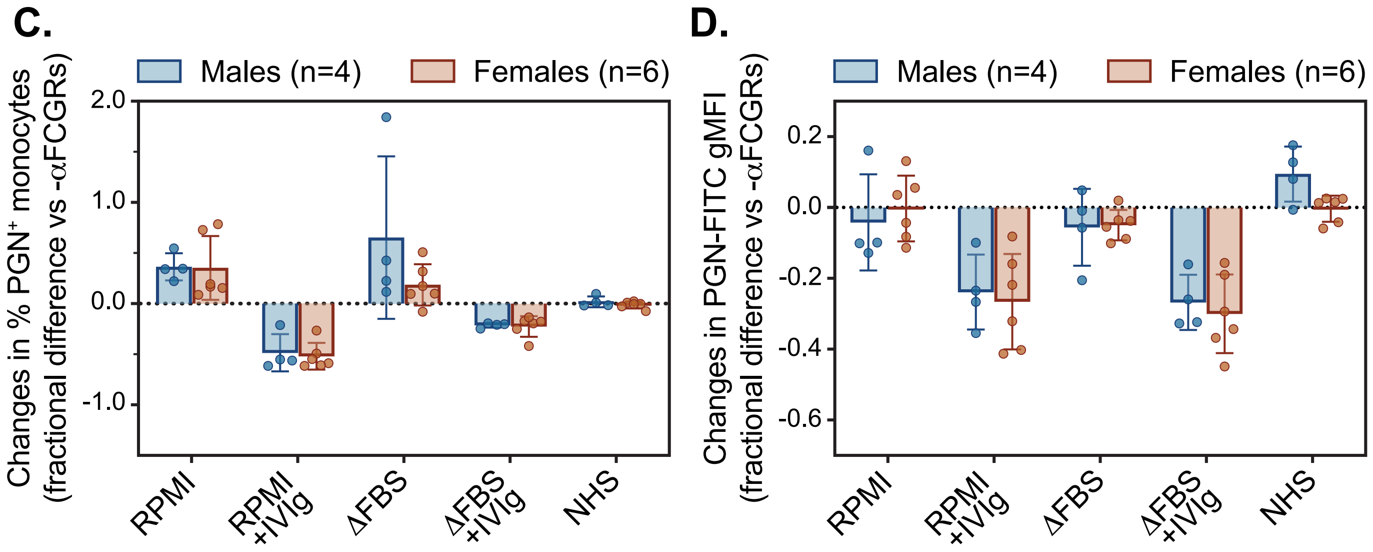

Supplemental Figure 1. The role of FCGRs during PGN uptake by primary human monocytes (data related to Figure 1)**.** (**A**) Gating strategy used for Figures 1-4, exemplified using a donor shown in Figure 1. (**B**) Representative flow cytometry histograms from a median responsive individual depicting PGN-FITC recognition and uptake under the opsonization conditions noted above panels, in the absence (top row, - αFCGRs) or the presence of FCGRs neutralization (bottom row, + αFCGRs). (**C**, **D**) Sex stratification of the data shown in Figures 1B and 1D respectively. Biological responses to opsonins and FCGR neutralization were not statistically different between the 2 sexes (2-way ANOVA with Holm-Šídák correction for multiple comparisons).

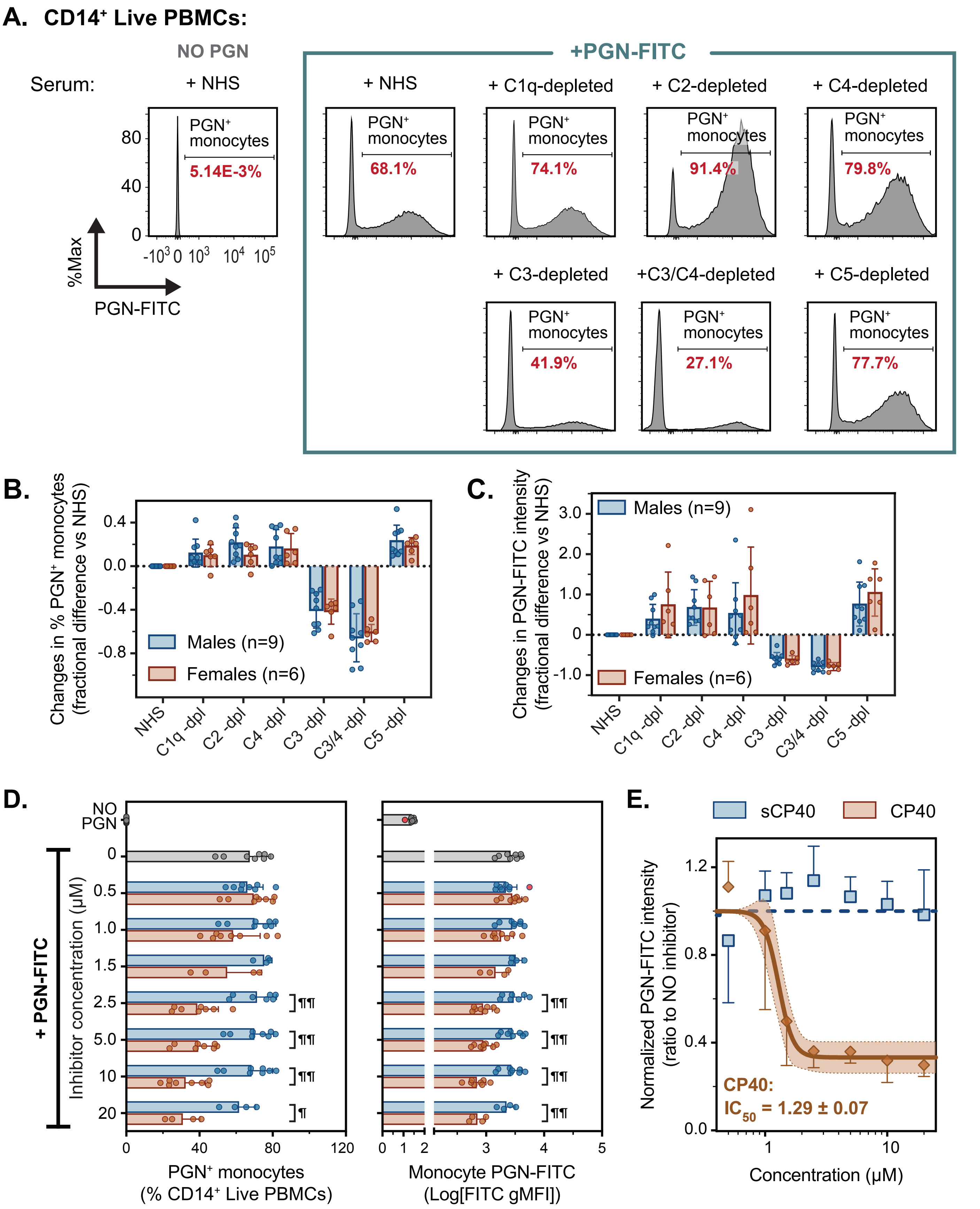

(Supplemental Figure 2 continues next page)

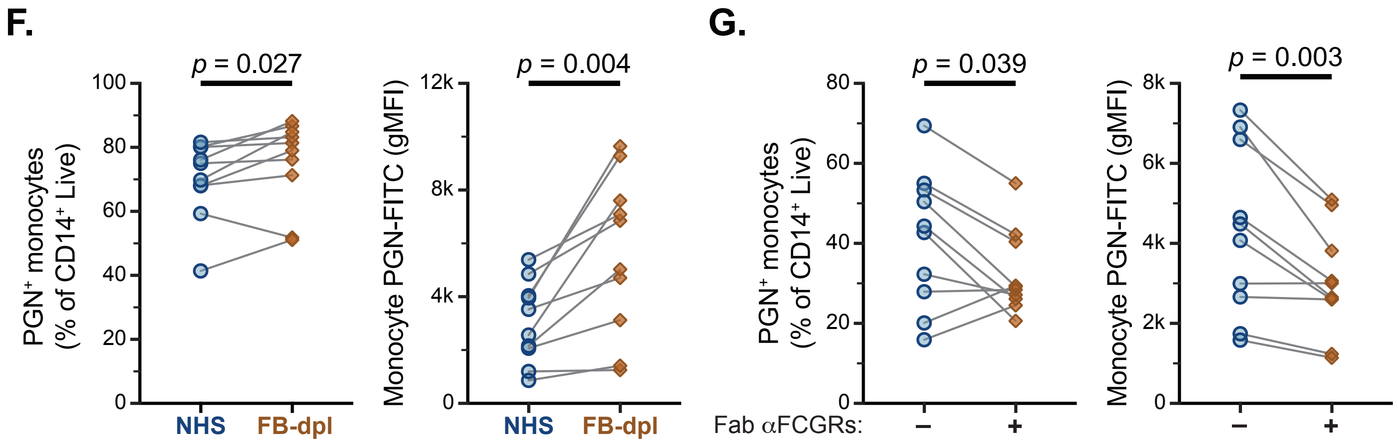

Supplemental Figure 2. Optimal PGN uptake requires opsonization by complement C3 (data related to Figure 2)**.** (**A**) Representative histograms from a median responsive individual depicted in Figure 2, A-D. The gating strategy used is identical to Supplemental Figure 1. (**B**, **C**) Sex stratification of data shown in figures 2C and 2D, respectively. Biological responses after complement depletions were not statistically different between the 2 sexes (2-way ANOVA with Holm-Šídák correction for multiple comparisons). (**D, E**) Effect of CP40, an inhibitor of C3 convertases, on PGN-FITC recognition and uptake by human monocytes. FITC labeled PGN particles were preopsonized in the presence of increasing amounts of CP40, or a scrambled peptide control (sCP40), before incubation with PBMCs. Data depict individual (n=8) and group averages ±SD (bars and whiskers) for the frequency of PGN$^+$ monocytes (**D**, left panel) and monocyte PGN-FITC intensities (**D**, right panel; log-transformed FITC gMFI). Differences between groups were assessed by a mixed-effects model with Holm-Šídák correction for multiple comparisons (**¶,** $P < 0.001$; and **¶¶,** $P < 0.0001$). After ratiometric normalization to uptake in the absence of inhibitor, the mean PGN uptake at increasing CP40 concentration was used to model the inhibitory capacity of CP40 (**E**). The best fit 4PL curve (continuous line) and the 95% confidence interval (shaded area) are shown, with the estimated IC_{50} highlighted in the insert. (**F**) Effect of ablation of the alternative complement pathway, through depletion of factor B (FB), on PGN-FITC recognition and uptake. Data depict paired comparisons between PGN uptake after opsonization with NHS or factor B depleted sera (FB-dpl). Differences between groups were assessed by Wilcoxon matched-pairs signed rank test (left panel) or a paired 2-tailed *t* test (right panel). (**G**) Effect of FCGR neutralization on PGN-FITC recognition and uptake after opsonization with C3/C4 depleted sera. Data depict paired comparisons between PGN uptake in the absence (–) or presence (+) of neutralizing Fabs against all FCGRs. Differences between groups were assessed by paired 2-tailed *t* tests.

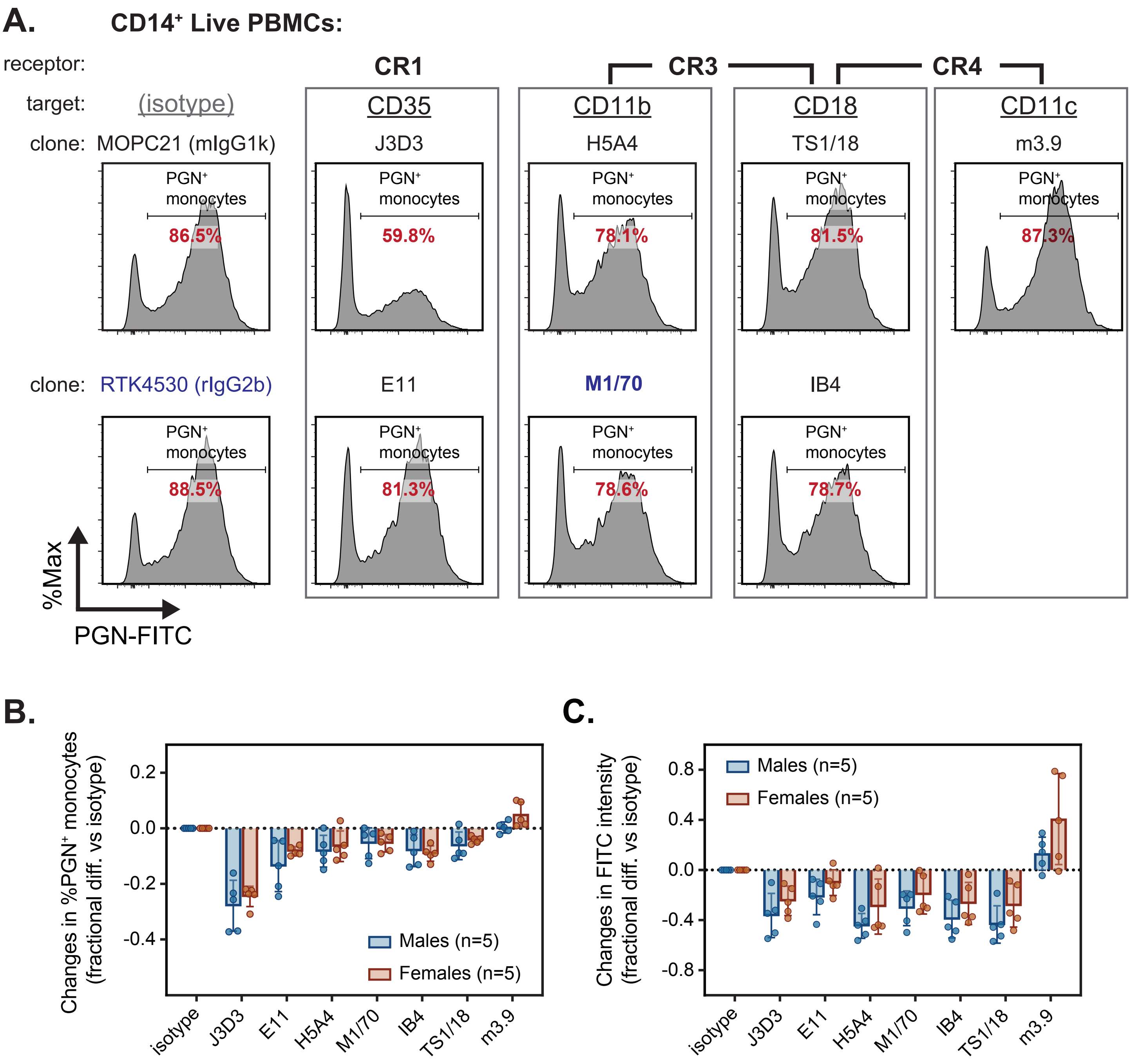

Supplemental Figure 3. Monocyte opsonophagocytosis of polymeric PGN is mediated by complement receptors CR1 and CR3 (data related to Figure 3)**.** (**A**) Representative histograms from a median responsive individual depicted in Figure 3. The gating strategy used is identical to Supplemental Figure 1. There were no significant differences between the two isotype controls tested, the MOPC21 mouse IgG1k (mIgG1k) and the rat RTK4530 IgG2b (isotype for the M1/70 monoclonal against CD11b). To reduce panel complexity, only results for MOPC21 are shown in Figure 3, while RTK4530 was omitted. (**B**, **C**) Sex stratification of data shown in Figures 3C and 3D, respectively. Biological responses after inhibition of complement receptors were not statistically different between sexes (2-way ANOVA with Holm-Šídák correction for multiple comparisons).

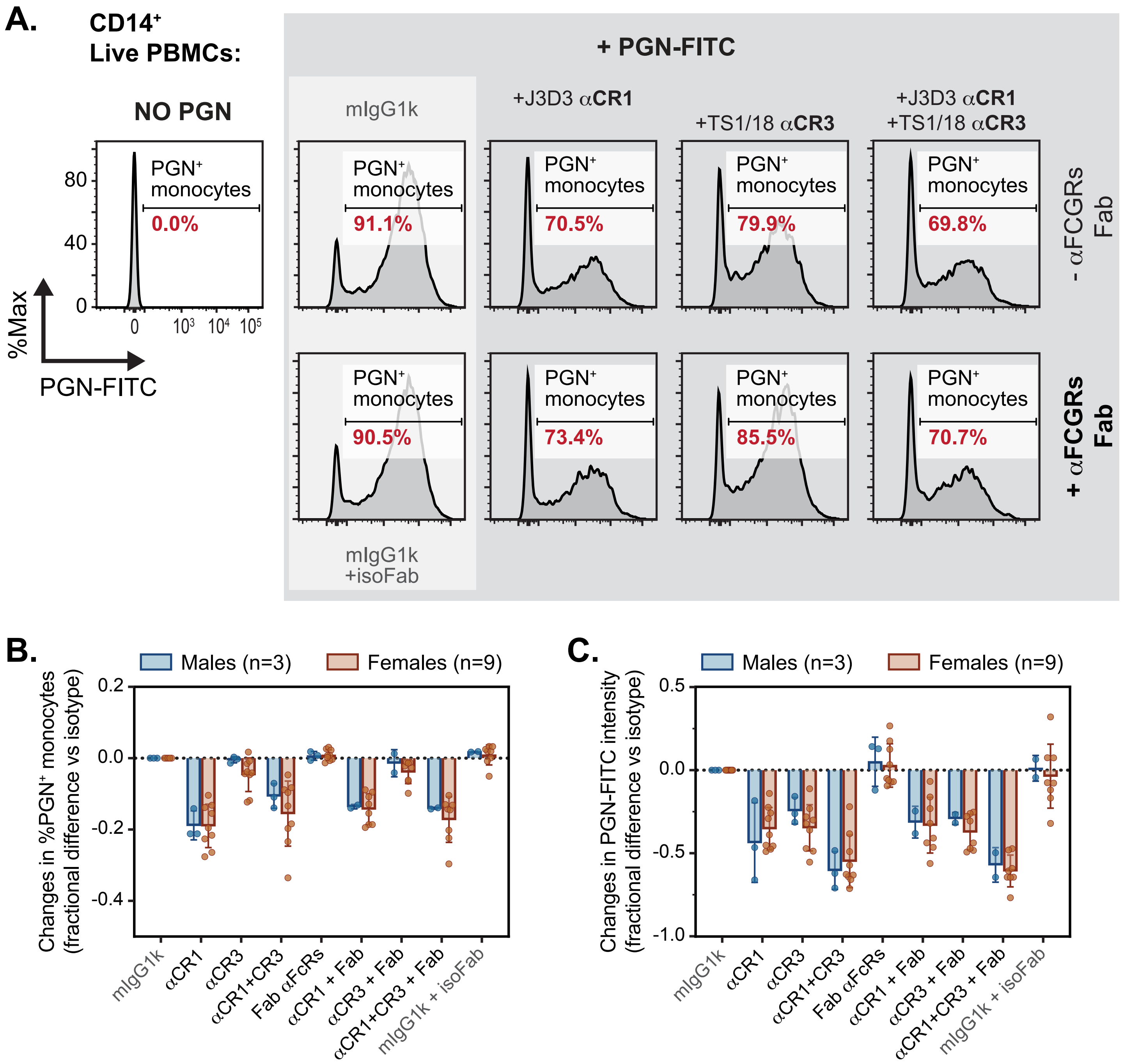

Supplemental Figure 4. Overlapping roles for CR1 and CR3 during monocyte opsonophagocytosis of polymeric PGN (data related to Figure 4)**.** (**A**) Representative flow cytometry histograms from a median responsive individual shown in Figure 4, depicting PGN-FITC recognition and uptake in the presence of inhibitory antibodies against CR1, CR3 and FCGRs, used by themselves or in combination. The gating strategy is identical to Supplemental Figure 1. (**B, C**) Sex stratification of data shown in Figures 4C and 4D respectively. Biological responses after inhibition of the opsonophagocytic receptors were not statistically different between sexes (mixed-effects model with Šídák correction for multiple comparisons, and individual variances computed for each comparison).

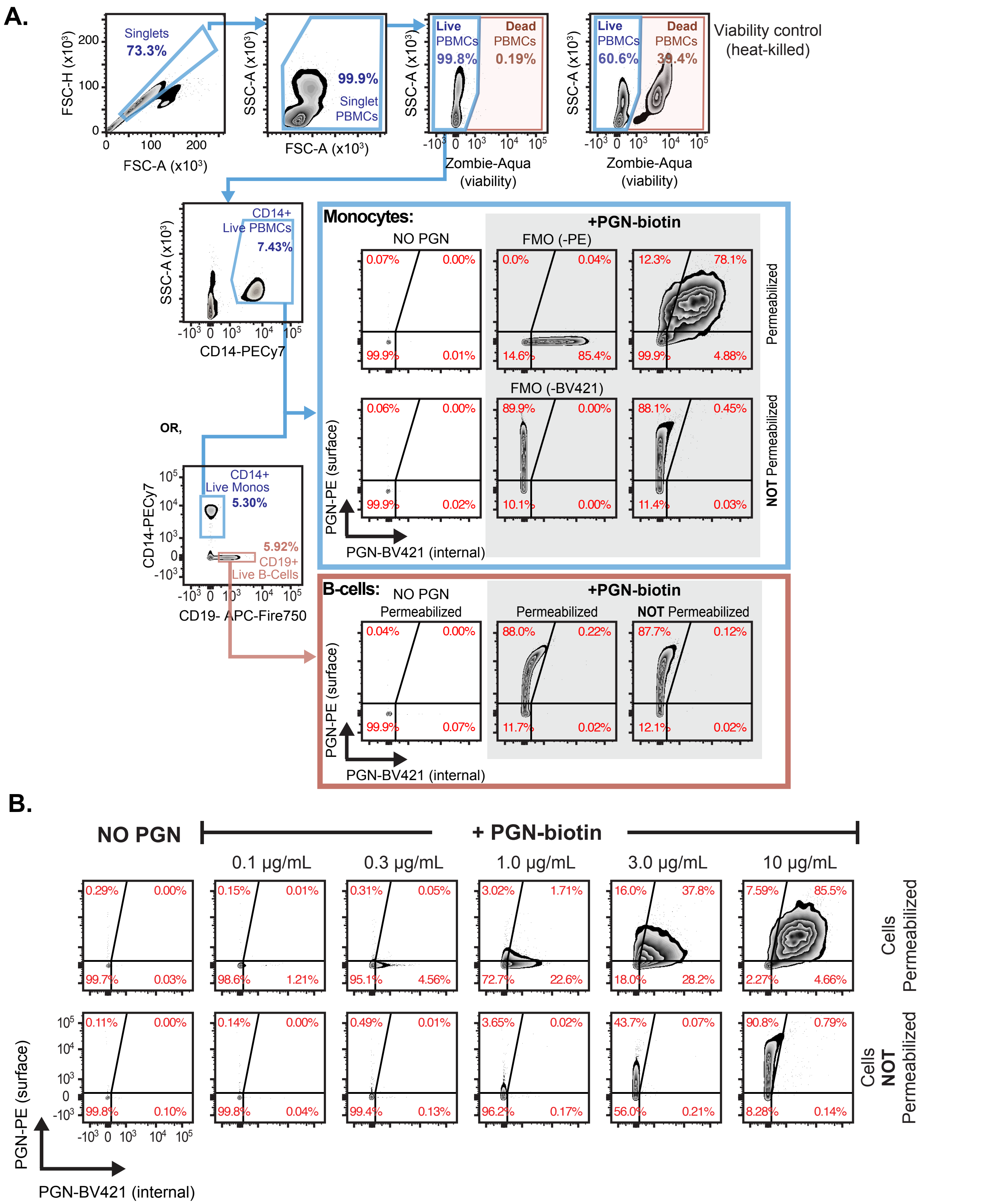

(Supplemental Figure 5 continues next page)

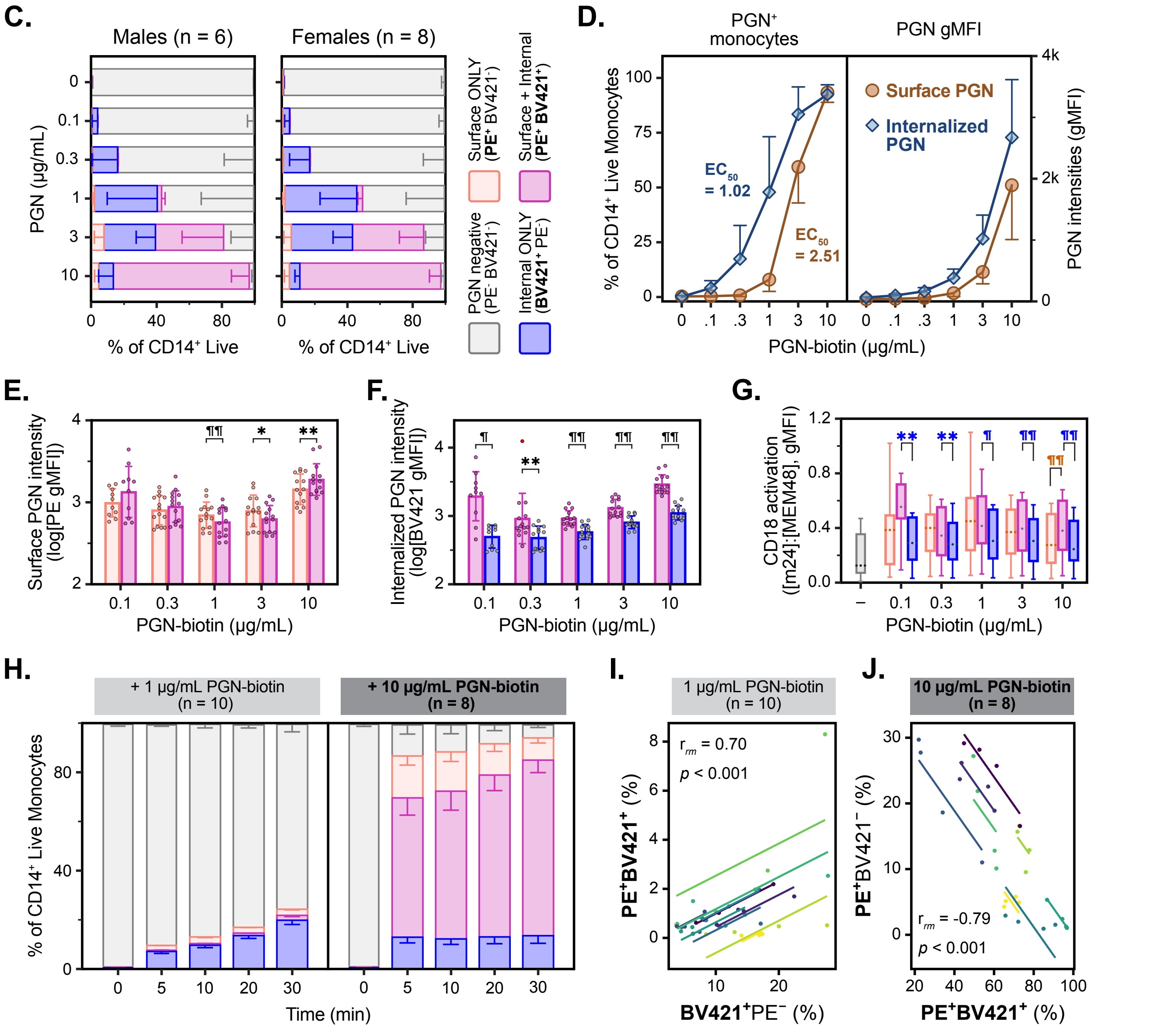

Supplemental Figure 5. Dose- and time-dependent dynamics of PGN recognition and uptake by primary human monocytes (data related to Figure 5)**.** (**A**) Gating strategy used for Figures 5 and 6. (**B**) Biaxial plots from a representative individual depicted in Figure 5B. Surface and internalized PGN-biotin was sequentially stained with PE-labeled streptavidin before, and BV421-labeled streptavidin after, cell permeabilization (top row). Staining in the absence of permeabilization performed in parallel (lower row) was used for background correction for the internalized PGN. (**C**) Sex stratification of data depicted in Figure 5B. Stacked bars depict mean (± SD) frequencies of monocyte subsets containing surface (PE+) and/or internalized (BV421+) PGN. Donor sex had no statistically significant impact on the relative distribution of monocyte subsets. (**D**) Dose-dependent changes for surface (PE) and internalized (BV421) PGN. Group averages ± SD (n= 14) are shown for either the frequencies of PGN^{+} monocytes (left) or PGN intensities (right). The observed EC_{50} shown in inserts were calculated by a best fit asymmetric sigmoidal (5PL) curve and indicate a faster internalization of PGN at lower concentrations. (**E-G**) Characterization of PGN intensities and CR3 conformational activation in monocyte subsets containing surface (PE) and/or internalized (BV421) PGN. Datasets were analyzed by RM 2-way ANOVA with Holm-Šídák correction for multiple comparisons (*****, $P < 0.05$; ******, $P < 0.01$; **¶**, $P < 0.001$ and **¶¶**, $P < 0.0001$). (**H**) Time-dependent changes in the relative distribution of monocyte subsets during internalization of either 1 μg/mL (left, n=10) or 10 μg/mL (right, n=8) PGN-biotin. (**I, J**) Repeated measures correlation analysis between $BV421^{+}$ (**I**, 1 μg/mL PGN uptake) and PE^{+} monocyte subsets (**J**, 10 μg/mL PGN uptake) within the time course dataset depicted in panel H. Individual donor responses are depicted with the same color, and the repeated measures correlation coefficients (r_{rm}) and *P* values are shown in inserts.

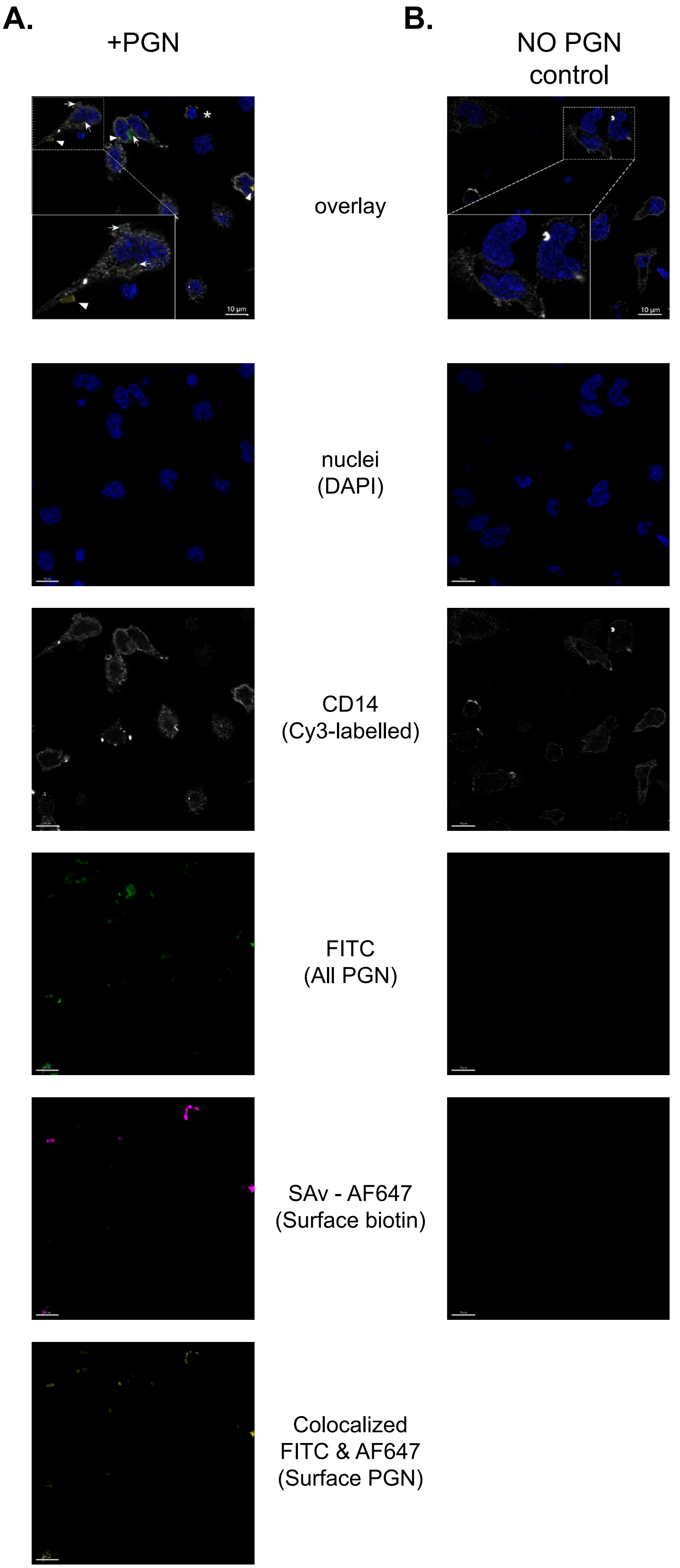

Supplemental Figure 6. Confocal imaging of PGN recognition and uptake of PGN by primary human mononuclear cells (data related to Figure 5C). PBMCs were captured on poly-L-lysine coverslips and incubated with 10 µg/mL biotinylated PGN-FITC particles for 30 min at 37°C, then fixed and stained as detailed in the Methods section. Surface-exposed biotinylated PGN-FITC particles were stained with AlexaFluor 647-conjugated streptavidin (magenta), and the colocalized fluorescent signals were assessed in Imaris (yellow, arrowheads). Internalized PGN-FITC particles were protected from streptavidin staining and detected as FITC fluorescence only (green, arrows). Monocytes were detected with an anti-CD14 monoclonal followed by a Cy3-conjugated secondary (white). (**A**) Overlay (top) and individual channel fluorescence signals for the micrograph depicted in figure 5C. (**B**) Representative micrographs from the paired negative control, untreated PBMCs from the same donor.

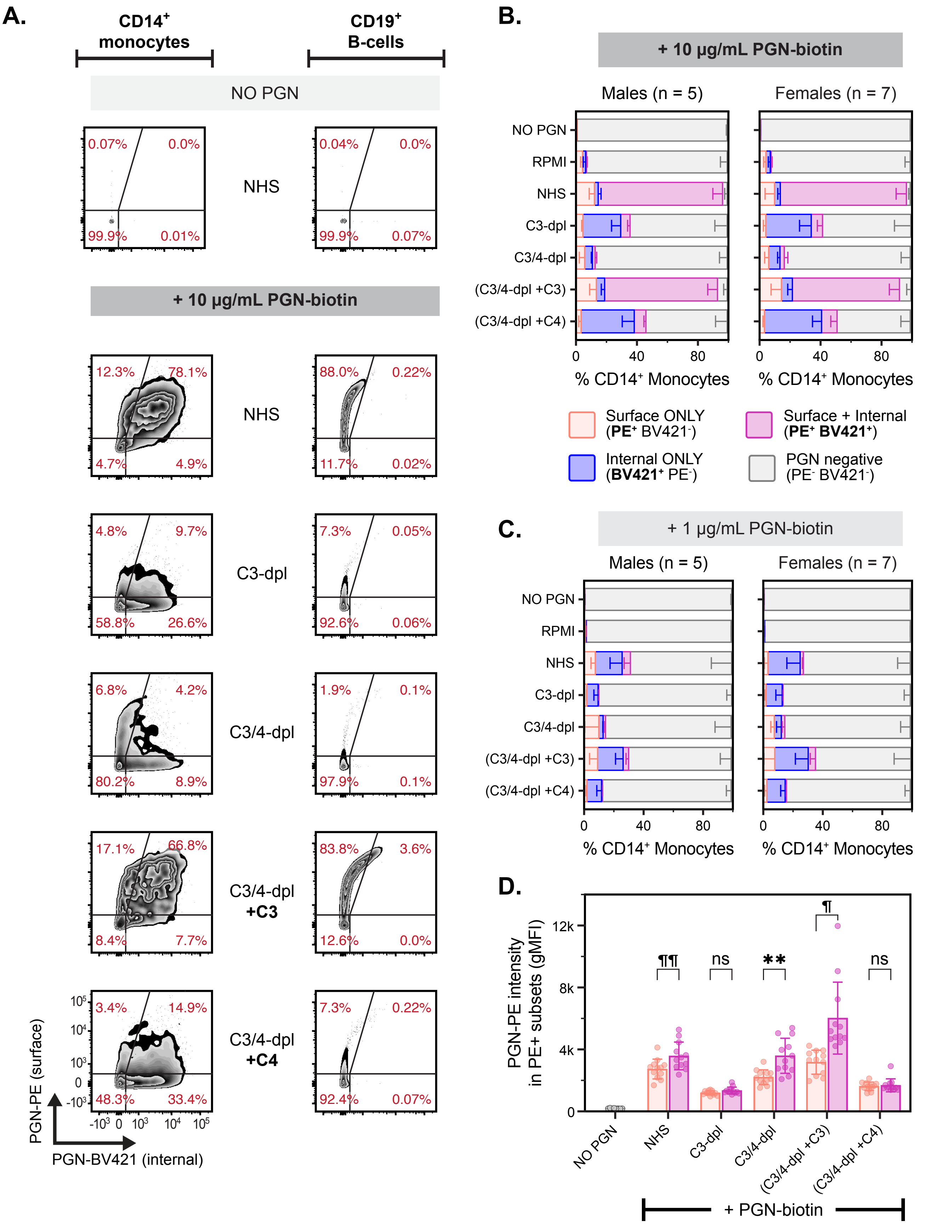

(Supplemental Figure 7 continues next page)

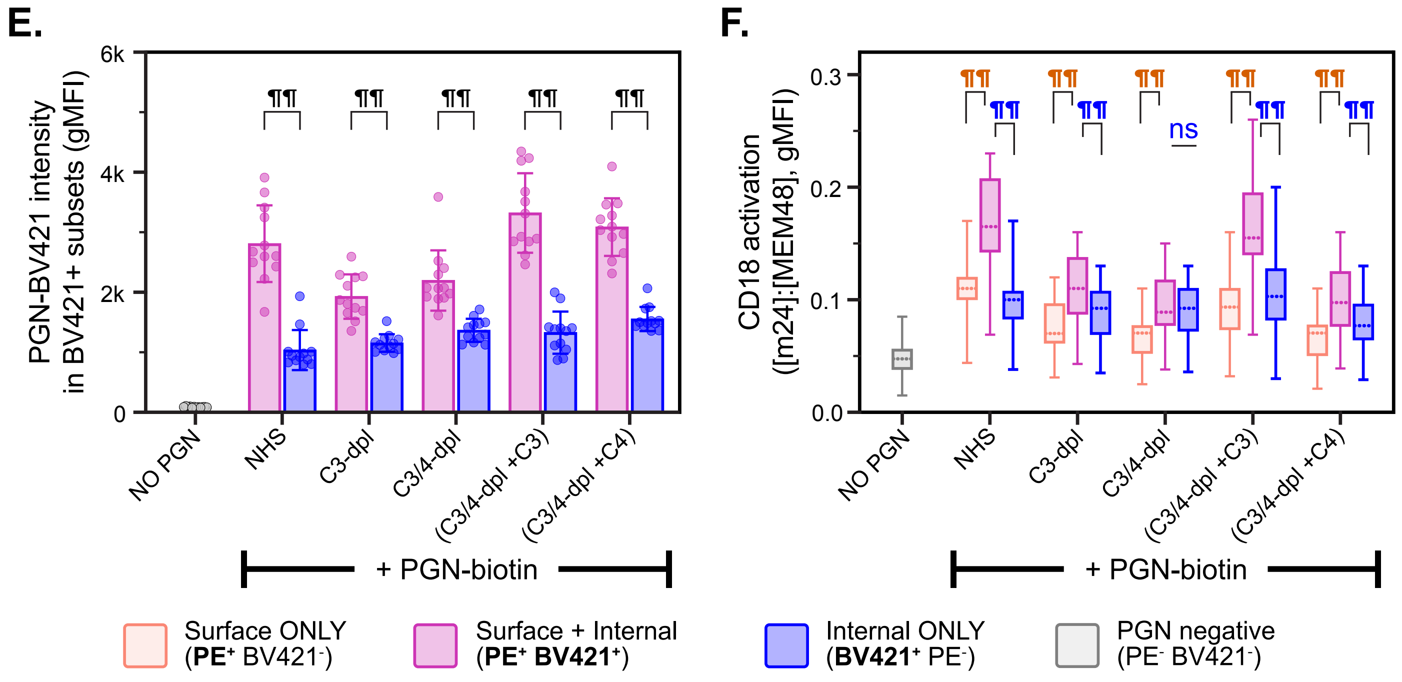

Supplemental Figure 7. Opsonin requirements for PGN recognition and uptake by primary human mononuclear cells (data related to Figure 5, E-F)**.** (**A**) Pairwise comparison of opsonin requirements for the recognition and uptake of biotinylated PGN by primary human monocytes ($CD14^+$, left column) and B cells ($CD19^+$, right column) from a representative individual depicted in Figure 5E. (**B**, **C**) Sex stratification of data shown in Figure 5F, depicting the relative distribution of monocyte subsets after uptake of PGN-biotin at either 10 µg/mL (**B**) or 1 µg/mL (**C**) in the presence or absence of C3 and C4 opsonins. Stacked bars represent mean± SD frequencies of monocyte subsets containing surface (PE^+) and/or internalized ($BV421^+$) PGN. Donor sex had no significant contribution to the relative distribution of monocyte subsets in either dataset. (**D-F**) Characterization of the monocyte subsets containing surface (PE^+) and/or internalized ($BV421^+$) PGN after uptake of 10 µg/mL PGN-biotin. In the presence of C3 opsonin, the intensity of surface PGN-PE staining was significantly higher in PE^+BV421^+ monocytes compared to PE^+BV421^- monocytes (**D**), suggesting additional engagement of C3b receptors during progression from PGN^- to PE^+BV421^- to PE^+BV421^+ monocytes. The intensity of the PGN-BV421 staining was always higher in PE^+BV421^+ monocytes compared to single $BV421^+PE^-$ across all conditions tested (**E**), indicating enhanced internalization of PGN in the PE^+BV421^+ subset. (**F**) Conformational activation of CR3, quantified as the ratio between the activation-dependent m24 mAb and the activation-insensitive MEM48 (see also Figure 6E), was higher in PE^+BV421^+ cells compared to either single PE^+BV421^- or $BV421^+PE^-$ monocytes, suggesting CR3 engagement during progression towards double positive monocytes (PE^+BV421^+), which was dependent on C3. (**D**, **E**) Data depict group averages ± SD (bars and whiskers, n= 12) and individual geometric means of fluorescent intensities (gMFI). (**F**) Data depict the interquartile range (floating bars), group medians (dashed line), and ranges (whiskers), while individual responses were omitted to aid visualization. Datasets were analyzed by RM 2-way ANOVA with Holm-Šídák correction for multiple comparisons, and selected comparisons of interest are highlighted graphically (ns, not significant; ****,** P < 0.01; **¶,** P < 0.001, and **¶¶,** P < 0.0001).

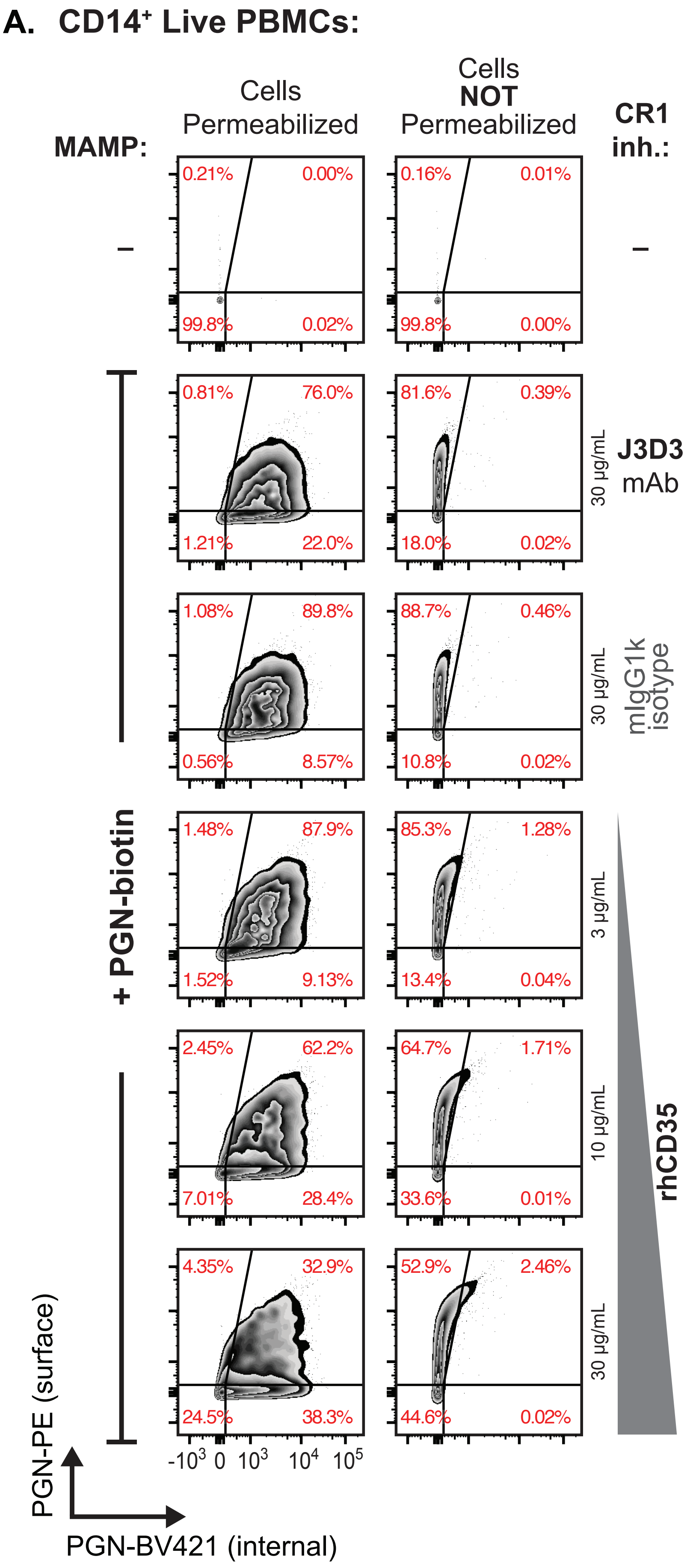

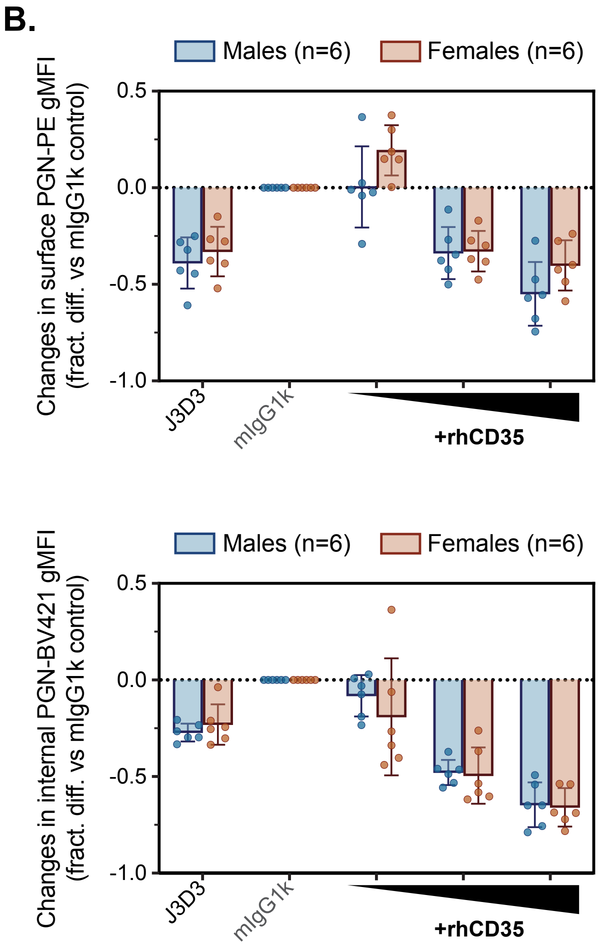

Supplemental Figure 8. Soluble CR1 (rhCD35) reduces both recognition and internalization of polymeric PGN (data related to Figure 6)**.** Representative biaxial plots (**A**) from a median responsive individual shown in Figure 6, illustrating the recognition and uptake of 10 µg/mL biotinylated PGN in the presence of CR1 inhibitors, either the J3D3 monoclonal or increasing concentrations of the extracellular domain of recombinant human CR1 (rhCD35). Surface and internalized PGN pools were sequentially stained with PE-labeled streptavidin before, and BV421-labeled streptavidin after, cell permeabilization (left column). Staining in the absence of permeabilization performed in parallel (right column) was used for background correction for the internalized PGN. (**B**) Sex stratification of data shown in Figure 6D illustrating normalized changes in surface (top panel) and internalized (bottom panel) PGN intensities after CR1 inhibition with the J3D3 mAb or increasing concentrations of rhCD35. Biological responses were not statistically different between the sexes (2-way ANOVA with Holm-Šídák correction for multiple comparisons).
